# Attention-grabbing news coverage: Violent images of the Black Lives Matter movement and how they attract user attention on Reddit

**DOI:** 10.1371/journal.pone.0288962

**Published:** 2023-08-09

**Authors:** Theresa Henn, Oliver Posegga

**Affiliations:** Department of Information Systems and Social Networks, University of Bamberg, Bamberg, Germany; ’Enrico Fermi’ Research Center, ITALY

## Abstract

Portrayals of violence are common in contemporary media reporting; they attract public attention and influence the reader’s opinion. In the particular context of a social movement such as Black Lives Matter (BLM), the portrayal of violence in news coverage attracts public attention and can affect the movement’s development, support, and public perception. Research on the relationship between digital news content featuring violence and user attention on social media has been scarce. This paper analyzes the relationship between violence in online reporting on BLM and its effect on user attention on the social media platform Reddit. The analysis focuses on the portrayal of violence in images used in BLM-related digital news coverage shared on Reddit. The dataset is comprised of 5,873 news articles with images. The classification of violent images is based on a VGG19 convolutional neural network (CNN) trained on a comprehensive dataset. The results suggest that what significantly affects user attention in digital news content is not the display of violence in images; rather, it is negative article titles, the news outlet’s political leanings and level of factual reporting, and platform affordances that significantly affect user attention. Thus, this paper adds to the understanding of user attention distributions online and paves the way for future research in this field.

## Introduction

It is indisputable that “[t]he mainstream adoption of social media applications has caused a paradigm shift in how people communicate, collaborate, create, and consume information” [1]. This paradigm shift has also affected the consumption of and confrontation with news [2], as users are increasingly “reading the news through social [media] platforms” [3]. During the last ten years, social media platforms have risen to one of the most prominent sources for news consumption [3,4], with nearly half of the U.S. population receiving news from social media websites in 2021 [5]. Thus, it becomes increasingly important to understand how news attract user attention in a social media context since the exposure to news shared on social media can have severe offline consequences [6], and news shared online is an integral part of understanding today’s media system [7,8]. The analysis of news shared online has thus become an essential precursor to improving our understanding of how social media news consumption influences “civic awareness and engagement in civic and political life” [9].

With this paper, we aim to contribute to this line of research by analyzing how the *violent* coverage of social movements affects user attention on social media. The portrayal of violence in traditional media types such as “television, radio newspapers, magazines … or in other words, any form of mass communication available before the advent of digital media” [10] has been studied thoroughly [11,12]. Research finds that traditional media frequently feature violent content as a way of entertaining their audiences and, thus, attracting public attention [11–13], which yields greater revenues [14,15]. It remains uncertain, though, whether this link between the portrayal of violence and the attraction of attention can also be transferred to the online sphere. Initial analyses concluded that sharing images featuring violence by individual users on social media did not lead to greater user engagement [16,17], but the effects of online news featuring violence on social media user attention remain unclear. Therefore, we see the need to further our understanding of the relationship between violence featured in the digital news content shared on social media platforms and its effects on user attention.

To address this research gap, we analyze the portrayal on social media of the social movement Black Lives Matter (BLM) and how that attracts user attention. According to Diani [18], a movement can be defined as a social movement if it’s based on “networks of informal interactions between a plurality of individuals, groups and/or organisations, engaged in a political and/or cultural conflict, on the basis of a shared collective identity.” We characterize BLM as a social movement because it is a decentralized structure of networked individuals [19] fighting for political, structural, and cultural change [19] while collectively identifying and organizing under the umbrella of symbols such as the hashtag #BlackLivesMatter [20].

The BLM movement began with a social media hashtag after the acquittal of the man charged in the shooting death of Black teenager Trayvon Martin in Florida in 2012 [21], grew nationally in 2014 after the deaths of Michael Brown in Missouri and Eric Garner in New York [19], and was reignited by the death of George Floyd, a Black man murdered by a white police officer on May 25, 2020, in Minneapolis [21]. People all over the world went into the streets to show solidarity and join the movement’s fight against “racial injustice and police brutality” [21].

We analyze the news coverage of this specific social movement for two reasons. First, the BLM movement gained a considerable amount of media attention that “delegitimized and racialized the movement by accentuating conflict and violence” [22], which aligns with an established line of research that stresses that media coverage often focuses on violent and disruptive aspects of a movement’s protest rather than its goals and social criticism [22–25]. Since public opinion and support of a social movement depend highly on how that movement is portrayed in news reporting [26–28], especially when violence is involved [23], it is particularly interesting to analyze the news portrayal of the BLM movement, as both protestors and police have committed acts of violence [29]. Second, the BLM movement “has deep ties to social media” [21], with hashtags such as #BlackLivesMatter sparking controversial online discussions and fueling worldwide protests and political unrest [21]. The consequence of the public being drawn to violent media content online could be a negative public bias toward the movement, which may escalate the tense political situation surrounding that movement [29].

In particular, we examine the effect of images that depict violence and nonviolence and are used in news articles shared on the social media platform Reddit, as images play a significant role in how news articles shape “the public’s understanding of issues and events” [30] (for a more detailed description of subreddits and the types of submission we analyze, see section “Social media platform Reddit”). These images are either photos or still images from videos that are part of the news articles linked in submissions posted on Reddit (henceforth, we refer to both as “images”). These linked news articles are either hosted by the digital extension of traditional media formats, such as on the website of the *New York Times* newspaper or are hosted by digital-born media, such as blogs or pure online news outlets [31,32].

As it is unclear whether earlier findings from media research can be transferred to social media environments [33], we focus not only on the effects on user attention of images featuring violence but also evaluate the impact of other attributes of shared digital news content that research has shown to be significant predictors of user engagement (for more information, see section “Theory and hypotheses”). We structure these attributes into three categories: (1) attributes related to the news item (in our case, the submission featuring a linked news article), such as the sentiment expressed in a news article’s title; (2) attributes of the news provider (in our case, the digital extension of traditional media formats or digital-born news media), such as the outlet’s political leanings or level of factual reporting; and (3) attributes concerning the community rules and platform affordances of the subreddits analyzed, such as the flagging of submissions that violate submission guidelines. By considering this multitude of attributes that can affect user attention, our analysis accounts for the complexity of social media landscapes, with their various, mutually dependent elements [1].

As our objective is to elucidate whether the portrayal of violence or other attributes of digital news content shared on Reddit have a significant impact on user attention, we pose the following two research questions specific to BLM, which is the social movement on which we focus in our analysis:

*RQ1*: *How does the visual portrayal of violence in BLM-related news articles shared on Reddit affect user attention*?
*RQ2*: *How do other attributes of digital BLM-related news content shared on Reddit impact user attention*?

Thus, our paper contributes to the understanding of the link between the type of online news coverage (violent and nonviolent) of a popular social movement [29] and the attention paid to it on the social media platform Reddit. By analyzing several attributes of online news reporting that impact user attention, our work not only tests the effect of images featuring violence on user attention but also accounts for attributes related to the news item, news provider, and community rules and platform affordances that might influence user attention. Since news consumption through social media continues to grow [3,4], this work contributes to the understanding of news consumption in an increasingly digitized world.

This paper is structured as follows. First, we briefly summarize the main steps and findings of the research to highlight our study’s contribution. Second, we dive deeper into previous research analyzing the relationship between news coverage of social movements and attention distributions while differentiating between traditional, digital, and social media. Third, we present our theoretical background and hypotheses in detail, grounded in the “negativity bias” and the “picture superiority effect.” Fourth, we detail our methodology, including the operationalization of our key variables, a description of our data collection on Reddit, and the transfer learning and performance of the VGG19 convolutional neural network (CNN) that we trained to classify violence in images. After conducting our data analysis, we discuss our findings, highlight the limitations of our work, and provide an outlook for future research.

## Summary of main steps and findings

The paper’s main goal is to analyze the relationship between news images that feature violence and user attention in the context of BLM on Reddit (RQ1), as there has been only scarce research on the relationship between digital news content featuring violence and user attention on social media. Based on the theoretical concepts “negativity bias” [34,35] and the “picture superiority effect” [36], we assume that there should be a positive effect between images featuring violence and user attention. In addition, we test for several other attributes of online news reporting that potentially impact user attention (RQ2) and subdivide them into (1) attributes related to the news item, (2) attributes of the news provider, and (3) attributes concerning community rules. For all attributes, we theoretically derive and test hypotheses empirically (Table 1 is an overview of the hypotheses and their acceptance or rejection).

**Table 1:** Overview of hypotheses and their acceptance or rejection.

Hypotheses		Acceptance	Rejection
*H1*: *Users will pay greater attention to submissions that feature images depicting violence than submissions that do not*.			X
*H2*: *A submission with a title conveying a negative sentiment will receive greater user attention than a submission with a positive title*.	X		
*H3*: *A submission’s linked image will have a more substantial effect on user attention than the sentiment of a submission’s title*.			X
*H4*: *A submission that includes a link to a conservative news outlet will receive greater user attention than a submission that includes a link to a liberal or neutral news outlet*.			X
*H5*: *A submission that includes a link to a less-factual news outlet will receive greater user attention than a submission that includes a link to a highly factual news outlet*.			X
*H6*: *A submission that includes a link to a popular news outlet with high traffic will receive greater user attention than a submission with a link to a less-popular news outlet with minimal or medium traffic*.			X
*H7*: *A submission posted in r/politics will receive greater user attention than a submission posted in r/news or r/worldnews*.			X
*H8*: *Users will pay greater attention to submissions flagged as “NSFW” than to nonflagged submissions*.			X
*H9*: *A submission with a flair tag will receive less user attention than a submission without a flair tag*.	X		
*H10*: *The number of a submission’s cross-posts will positively affect user attention*.	X		

For the empirical analyses, we proceeded as follows. We used the Pushshift submission dumps to download submissions from the three popular news-sharing subreddits, r/politics, r/news, and r/worldnews. We filtered the data to match our topic (BLM), period of analysis (May 25, 2020, the day of George Floyd’s murder, to May 25, 2021), and submissions that received a minimum of attention (removal of submissions with zero comments). To complete the dataset with variables necessary for testing our hypotheses, we undertook three methodological steps: (1) for the attributes of the news provider, we added information regarding the linked news outlet’s political leaning, factual reporting, traffic, and type of media outlet from the commonly used website mediabiasfactcheck.com, which is widely applied in research [37]; (2) for each submission, we downloaded, if possible, the first image of the linked news outlet and automatically classified it as violent or nonviolent with a VGG19 CNN that we trained on a comprehensive and context-specific dataset; and (3) to detect the sentiment of a news title (attribute related to the news item), we trained a Bidirectional Encoder Representation from Transformers (BERT) model and classified each title as either positive or negative. We received information on all other variables related to attributes of community rules from the Pushshift submission dumps. We conducted a negative binomial regression to analyze the relationship between user attention (measured in number of comments) and our independent and control variables that we derived theoretically from the existing literature.

Based on the results of our analysis, we can answer the two research questions. With respect to RQ1, it is not images featuring violence that affect user attention in the case of BLM reporting on Reddit. Rather, with respect to RQ2, we find that (1) the political leaning of a news outlet (politically neutral news outlets have a positive effect compared to conservative or liberal outlets); (2) its level of factual reporting (high factual reporting is positively associated, low factual reporting is negatively associated compared to mixed factual reporting); (3) Reddit community rules and affordances (the subreddits r/news and r/worldnews are positively associated compared to r/politics, link flair is negatively correlated, the number of cross-posts has a positive effect), and (4) the negative titles of news significantly correlate with user attention in the case of BLM reporting on Reddit.

In the following, we provide a thorough explanation of our theoretical background and methodological procedure and discuss our results in detail.

## Theoretical background

### Related literature

In the past, traditional media such as newspapers, television, and radio– known for their gatekeeping function, agenda-setting power, and one-way communication– have been the predominant source for news consumption [38]. The media system, however, has changed [8], and a digital transformation has led traditional media to enter the online space and distribute news through their own digital channels (e.g., the online version of *The New York Times*). These traditional types of media have been complemented by digital-born media such as blogs or pure online newspapers that “produce a wide variety of content, from in-depth investigative journalism to eyeball-catching clickbait” [8].

The introduction of social media networks such as Reddit, Twitter, and Facebook, which function as intermediaries for news exposure and consumption [39], is another addition to this growing digital side of the media system. On these platforms, digital news can be produced and shared by both individual users and large news organizations, emphasizing the significant shift in power structures in news production. Thus, social media networks “are becoming more and more integral to how people find information [and news]” [40] by also enabling people to produce, share, and discuss information and news with their networks of other people.

This interplay between digital media (comprised of the digital extensions of traditional media and digital-born media; henceforth referred to as “digital media”) and social media platforms are the focus of our analysis. Together with traditional media, they constitute the so-called “hybrid media system” [7].

The relationship between media and their reporting on social movements has a long history in research [27,28,41–44]. When reporting on social movements, both digital and traditional media outlets need to decide which events to include in their news coverage and how to portray the social movement [see, e.g., 27,41,42]. Since some events are regarded as more “newsworthy” than others [42], as they “resonate with more general social concerns” [43], media outlets report on specific events rather than others, which is also referred to as “selection bias” in media research [42,43]. A particular selection criterion in this context is the reporting on negative events, especially in a political or social movement context [see, e.g., 44,45], as negativity, in general, has long been prevalent in the news coverage of both traditional and digital media [46,47].

Reporting about violence is a specific type of negative news coverage [48]. Social movements, in particular, have been subjected to this type of negative news coverage [e.g., 49,50] “emphasizing violence and deviant behaviour” [22], which tends to increase the audience size as it “contributes to the media spectacle … around protest events” [16]. Yet, this delegitimizing news coverage and focus on violence that amplifies the sensationalist aspects of protest is “often to the detriment of the social cause or movement …” [26]; it is referred to in research as the “protest paradigm” [23,25]. Thus, media reporting constructs a certain interpretation of the protest event, also called “description bias” [see, e.g., 28], “that differs from both the objectives of protestors and interpretations of other observers” [28], which in turn highly impacts the public perception of the social movement [e.g., 23,25,26]. Therefore, it is important to further our knowledge of media reporting on social movements featuring violence and the attention it attracts.

To thoroughly understand the effects of violence in media reporting and its impact on attention, we need to differentiate between traditional and digital media news reporting. Whereas in a social movement context the role of violence in traditional media coverage has been studied thoroughly, though little attention has been paid to its effects in digital news reporting (see next paragraphs). Thus, our paper analyzes whether findings from traditional media research can be replicated for the digital news coverage.

The literature shows that traditional media’s news coverage frequently features violent aspects of social movements [see, e.g., 23,41,51] to attract attention, as “[b]urning buildings and burning tires make better television than peaceful vigils and orderly marches” [41]. For instance, Boykoff [52] showed that influential newspapers, in particular, typically apply framing that features violent aspects of social movements because it serves as a prominent device for attracting readers’ attention. McFarlane and Hay [51] evaluated the newspaper reporting on the protests against the 3^rd^ World Trade Organization (WTO) Ministerial Conference in 1999, which portrayed protests “… within an ‘anarchy and violence’ narrative structure in which repeated reference was made to property destruction by protestors, to their acts of violence, and to the presence of ‘masked anarchists dressed in black’” [51]. This particular framing was applied to resonate with the news’ readership and receive attention while delegitimizing the protest and guiding “readers towards a positioning supporting the WTO” [51].

In a similar vein, in his study about the press reporting on the 1968 London anti-war rally, Murdock [50] showed that the press framed the movement to a viewpoint “already familiar to the reader” [50] by portraying the protestors as militant extremists or anarchists ready to use violence. This framing made the news coverage more appealing to the audience and therefore added to its received attention. Likewise, Boyle et al. [23] found that newspapers often emphasize the violent actions of social movements disproportionally when reporting on them, regardless of country and across time. Such violent framing of a movement “can have a powerful influence on how audience members perceive issues and parties to a conflict, including perceptions of protestors” [23]. McLeod and Detenber [53] were among the first to analyze the impact of television coverage on violent protests and the effects on public identification with a movement. The authors showed that news stories shared on television have the power to legitimize or delegitimize the cause that motivates protest and thus sway the public in favor of or against a social movement. Vliegenthart and Walgrave [54] summed up the findings of several different authors who concluded, among other findings, that a social movement protest is more likely to receive traditional media coverage if it includes acts of violence; this leads to greater audience exposure and thus greater attention.

The notion of violence and social movements also plays a role on social media. Some studies have analyzed how activists themselves use violence in their social media posting (tweets, videos, and photos) as a means of drawing attention to their cause [55]. Since the sharing of images affects user attention online [22,56], and such images are decisive for how social movements are perceived [57,58], it is important to analyze the visual portrayal of violence in the social media communication of social movements.

Neumayer and Rossi [59] contrasted how different actors, such as police, journalists, and activists, shared images with varying levels of violence on the social media platform Twitter as a way of communicating their issues. They concluded that activists often struggle to garner visibility for their grievances because images posted by other actors (e.g., the police) that feature violence overshadow the cause of the movement. Through a manual image content analysis on Twitter, Kharroub and Bas [17] found that in the 2011 Egyptian revolution, it was not emotionally arousing images (featuring, e.g., violence) posted by actors such as activists or the police that compelled users to retweet messages but rather efficacy-eliciting images (featuring, e.g., national or religious symbols). Rossi et al. [16] undertook the first large-scale study in this field, evaluating the correlation between images featuring violence posted by different types of users, such as activists, news agencies, and the police, and their propagation on Twitter during the G20 summit protests in Hamburg, Germany, in 2017. Within the five days of their study context, they found that the visual portrayal of violence did not affect the number of retweets or mentions.

In the aforementioned studies, the portrayal of violence on social media did not affect user engagement to the expected extent. These studies, however, evaluated images featuring violence that had been shared by various user types, such as activists, police, and journalists. Our analysis, by contrast, focuses exclusively on how digital *news coverage* of social movements and violence affects user attention on social media platforms. In addition, we control for other potential attributes of digital news reporting that could influence user attention. Thus, our paper offers three unique contributions. First, we evaluate whether previously established findings from traditional media research can be transferred to the online world, as research suggests with respect to offline news habits [60,61]. Thus, we test whether shared online news articles featuring visual displays of violence receive increased user attention on Reddit. Second, we analyze the case of a prominent and contemporary social movement with a strong digital presence over a prolonged period of time (one year) on the social media platform Reddit, which hosts subreddits with an explicit focus on sharing and discussing online news content. Third, since social media is a complex communication environment [1] and the effect of digital news coverage of violence on user attention is unclear, we extend our image analysis by analyzing other attributes of the shared digital news content (attributes of news item, news provider, and community rules and platform affordances). By doing so, we create an extended statistical model that accounts for several plausible factors that affect online user attention.

### Theory and hypotheses

So-called “negativity bias” is one broadly acknowledged concept used to explain people’s tendency to be drawn to negative news, which is attributed to a psychological remnant of human evolution [see, e.g., 35]. Rozin and Royzman [34] describe the concept as “a general bias, based on both innate predispositions and experience, in animals and humans, [which] give[s] greater weight to negative entities.” Hence, negative information is experienced more intensely, leaving a more persistent impression in people’s memories than positive information [62]. This mechanism partially explains why we are naturally drawn to negative [47] and also violent media content: our “brain is simply built with a greater sensitivity to unpleasant news” [Prof. John T. Cacioppo cited in 63]. This is, in part, an explanation for the predominance of negative news reporting in traditional media [64]. Based on these findings and the suggestion that offline news habits can be transferred to digital news consumption [60,61], we hypothesize that digital news content depicting violence in the context of social movements should also receive greater user attention when shared on social media.

Further, in line with the saying that “a picture is worth a thousand words,” we argue that the visual display of violence should garner even greater attention [30]. Pictures have a more significant impact on the human brain than textual representations of information, which can be absorbed more rapidly through visual stimuli; the content of images is more likely than text to be remembered [36]; and emotionally charged images, in particular, tend to attract greater attention than those lacking in emotion [65]. This phenomenon is also referred to as the “picture superiority effect” [36]. Social media platforms have increasingly become a “visual medium” [66], a sign of the growing importance of images in online contexts [67]. Plus, it has been shown that the willingness of social media users to share (and thus engage with) a digital news article about social movements increases once visuals are included [22]. Thus, we analyze the effect on user attention of images featuring violence since sharing images highly correlates with user attention online [22,56].

This paper defines violence as “intentionally caused or carelessly accepted damage to/destruction of property or the injuring/killing of people” [68]. By applying this strict definition of violence by Rucht [68], our analysis focuses on the display of physical violence and intentionally excludes other forms of violence, such as verbal assault. We decided on this relatively conservative definition of violence because we aim to measure the effect of “extreme” forms of violence, which we assume receives more user attention than more “minor” types of violence (see section “Operationalization of variables” for further information).

Based on this theoretical background, our first hypothesis is:

*H1*: *Users will pay greater attention to submissions that feature images depicting violence than submissions that do not*.

As stated before, we analyze the effects of other BLM-related news content attributes shared on Reddit. We focus first on attributes related to the news item. We examine submissions to subreddits that consist of the title of a news article and a link to a digital source news outlet (henceforth, we refer to both digital extensions of traditional media and digital-born media as “news outlet”) and conclude that each submission’s title is an integral part of a submission’s content and can, thus, affect user attention [69]. Because an article’s tone can influence users’ perceptions of and reactions to the content [70], we control for the sentiment of titles. Since previous research established a positive relationship between negative titles and news readership online [71], we also assume that negative titles will be more attractive than positive ones, which also aligns with the negativity bias. From this, we derive our second hypothesis:

*H2*: *A submission with a title conveying a negative sentiment will receive greater user attention than a submission with a positive title*.

In line with the picture superiority effect, Hessel et al. [67] have shown that “image features always outperform text features” in their effects on user attention (see also [22]). Therefore, we argue that the impact of images featuring violence outweighs the effect of titles conveying a negative sentiment. Thus, our third hypothesis states:

*H3*: *A submission’s linked image will have a more substantial effect on user attention than the sentiment of a submission’s title*.

Other critical attributes that might influence user attention can be related to the news provider. Thus, we evaluate the political leanings of the news outlet referenced by a submission. There is an extensive body of research analyzing the relationship between political ideology and susceptibility to the negativity bias [72,73]. It is argued that people who identify as “conservatives” (as understood in the U.S. context) are more receptive to negativity compared to those who identify as liberals (as understood in the U.S. context) [72], although some recent studies have challenged these findings [73]. Based on these considerations, we assume that conservative news outlets cover greater amounts of violent and negative content than other outlets since they align with their audience’s expectations of news reporting [74] (openness to negative news). In line with the negativity bias, we assume that this type of reporting should lead to a higher level of user attention compared to liberal or neutral reporting. Hence, our fourth hypothesis is:

*H4*: *A submission that includes a link to a conservative news outlet will receive greater user attention than a submission that includes a link to a liberal or neutral news outlet*.

Likewise, a news article’s level of truthfulness has a major effect on user engagement and attention on social media. Research has shown that false news, in particular, “diffused significantly farther, faster, deeper, and more broadly” [75] on social media, thus reaching a broader audience and receiving greater user attention. Based on this discrepancy between true and false news, we assume that a news outlet’s level of factual reporting will influence user attention. Thus, news outlets that are known for a less-factual reporting style (“false news”) will receive greater user attention than outlets that adhere to a more factual style of reporting (“true news”). Our fifth hypothesis is:

*H5*: *A submission that includes a link to a less-factual news outlet will receive greater user attention than a submission that includes a link to a highly factual news outlet*.

Moreover, a news outlet’s general level of prominence could affect the level of attention a user pays to a submission that references it. Therefore, we also test for the linked news outlet’s traffic by assuming that those with more traffic are more popular and thus receive greater user attention. Hence, our sixth hypothesis states:

*H6*: *A submission that includes a link to a popular news outlet with high traffic will receive greater user attention than a submission with a link to a less-popular news outlet with minimal or medium traffic*.

Finally, we examine attributes related to Reddit-specific community rules and platform affordances “that shape how people engage with [online] environments” [76] and thus could have an effect on a submission’s visibility and, in turn, user attention. In our analysis, we evaluate data from the three subreddits r/politics, r/news, and r/worldnews. Of these, only r/politics allows the posting of thumbnail images, and so we assume–in line with our assumptions regarding the picture superiority effect–that submissions in this subreddit receive greater attention than submissions posted in the other two (for more information regarding the subreddits analyzed, see section “Social media platform Reddit”). By controlling for the subreddit in which a submission was posted, we also account for different community sizes, and that posting in more popular subreddits could lead to greater exposure and, therefore, greater user attention–or, in contrast, to less visibility due to greater competition for attention with other posts [69].

We also include whether a submission is flagged as “Not Safe for Work (NSFW),” which, in our context, likely implies the depiction of a higher degree of (physical) violence within the attached news link. Based on our previous assumptions regarding the effect of violence on user attention, we expect a positive relationship between “NSFW” submissions and user attention.

Further, we add whether a submission receives what Reddit calls a “flair tag,” which, in our case, indicates that the subreddit’s moderators commented on the submission. Considering that our three subreddits are known to follow strict submission guidelines (see the front page of each subreddit), we assume that submissions with a flair tag have somehow violated the submission guidelines by, for example, being out of date or irrelevant to the subreddit. Based on these considerations, we assume that submissions with a flair tag receive less user attention.

Finally, we control how often a submission has been cross-posted, that is, posted as a new submission to other subreddits. It seems plausible to assume that exposure to a broader audience also attracts greater user attention to the initial submission. We, therefore, assume a positive relationship between the number of cross-posts and user attention.

Summarizing these assumptions regarding the Reddit-specific affordances and community rules, our final four hypotheses are:

*H7*: *A submission posted in r/politics will receive greater user attention than a submission posted in r/news or r/worldnews*.
*H8*: *Users will pay greater attention to submissions flagged as “NSFW” than to nonflagged submissions*.
*H9*: *A submission with a flair tag will receive less user attention than a submission without a flair tag*.
*H10*: *The number of a submission’s cross-posts will positively affect user attention*.

We include additional control variables to control for contextual factors that could affect user attention online. We control for the timing and date of a submission, as Reddit is a dynamic environment with varying attention distribution over time [67]. Further, we test whether the type of news outlet referenced in a submission influences user attention. We differentiate between digital-born news outlets (websites) and the digital extension of traditional news media (e.g., TV stations and newspapers) since–generally speaking–both types of media likely display a different level of professionalism and follow a different logic of news reporting (see section “Related literature”), which might impact user attention differently.

## Materials and methods

### Operationalization of variables

Attention is a broad concept but can be defined generally as: “Notice taken of someone or something; the regarding of someone or something as interesting or important” [77]. There are several ways to interact with posts and “take notice” of a particular submission on social media. In the case of Reddit, we operationalize our dependent variable, “user attention,” with the number of comments posted on each submission. This proxy offers an indication of whether users interacted substantially with a submission [22] that seems to be particularly popular in a political news context [78] and thus paid attention to it. Other attention measures, such as up- or downvoting on a submission, can be seen as an expression of whether a submission’s content aligns with the community rules of a subreddit [79] and thus indicates quality control rather than being a measure of user attention. Therefore, we operationalize our dependent variable, “user attention,” with the discrete variable, “number of comments.” (To sustain our results, however, we also ran a negative binomial regression model that takes into account the number of up-and downvotes as an expression of user attention. Yet, also with this measure, the main effects remain the same. For more information, please see Supporting Information, Negative binomial regression models).

We use the conservative definition by Rucht introduced in section “Theory and hypotheses” to capture the complex concept of violence (*H1*). Hence, to be classified as “violent,” an image must show physical harm to property or people (in the case of the BLM movement, e.g., looting shops or using pepper spray against protestors). Images showing only “minor” violence, such as verbal assault or provocative gestures, are not coded as “violent.” As stated before, we intentionally apply this conservative definition of violence because, first, in the BLM context, there were significant acts of violence by protestors who looted shops and police officers attacking the protestors with “pepper spray, tear gas, and rubber bullets” [29]. Hence, we are optimistic about gathering enough meaningful data. We also anticipate that the conservative definition of violence will lead to more precise results by assuming that a more extreme visual display of violence attracts greater user attention. For our automated image classification, we dichotomously code a violent image as “1” and a nonviolent image as “0.”

As *H2* indicates, titles of submissions with negative connotations could positively impact user attention. To classify the connotation of each submission’s title as either “negative” or “positive,” we utilize a BERT model [80] as it depicts one of the most prominent and state-of-the-art language models for sentiment analysis [see, e.g., 81]. BERT is a transformer model that can capture the context of a sentence by considering the bidirectional dependencies between words within a sentence [80]. Thus, the model outperforms simpler approaches such as bag-of-word models that are dictionary-based and do not account for the context of the classified sentence [see, e.g., 81]. For our analysis, we use the “BERT base model uncased,” which we derived from the Hugging Face platform [82]. The model is trained on English Wikipedia and the BookCorpus data [80] and contains 110 million parameters. To customize the model for classifying the sentiment of news articles, we trained the model with the NewsMTSC dataset [83], a labeled dataset for sentiment classification of political news articles containing the categories positive, negative, and neutral. Since we are interested in positive and negative sentiment classification, we dropped the neutral samples from the training, validation, and test dataset. After training, the model receives a training accuracy of 99.17%, a validation accuracy of 87.66%, and a test accuracy of 87.40% (for more information on the data reduction, the training, and validation of the model, please see Supporting Information, Sentiment model). In addition, to evaluate the effect of sentiment on user attention even more granularly, we ran another negative binomial regression model with different sentiment variables, which, however, yields almost similar results (for more information, please see Supporting Information, Negative binomial regression models).

To label the categorical variables related to linked news outlets, we use information retrieved from the website mediabiasfactcheck.com, which is widely applied in research [37]. We manually label each news outlet’s “political leaning” (*H4*), “factual reporting” (*H5*), and “traffic” (*H6*) according to the respective classifications by mediabiasfactcheck.com. If we do not find all information on the news outlet, we code the observation as missing and drop the submission.

After coding our data, we merge some labels to avoid small *n* distributions within a category and obtain more sound results. Thus, our variable “political leaning” is comprised of the labels “liberal” (merging the “left,” “left-center,” and “far left” categories), “conservative” (merging the “right,” “right-center,” “far right,” “extreme right,” and “far right conspiracy pseudoscience” categories), “conspiracy” (merging the “conspiracy,” “junk news,” and “conspiracy pseudoscience” categories), and “neutral” (merging the “least bias” and “pro-science” categories). The variable “factual reporting” consists of the labels “high” (merging “high” and “very high”), “mixed” (merging “mixed” and “mostly”), and “low” (merging “low” and “very low”). Finally, the variable “traffic” is split into the labels “high,” “medium,” and “minimal.”

The remaining Reddit-specific community and affordance variables are coded as follows: the categorical variable “subreddit” (*H7*) is divided into the three subreddits, “r/politics,” “r/news,” and “r/worldnews”; both variables “NSFW” (*H8*) and “link flair” (*H9*) are binary and coded as “0” if the submission was not flagged and as “1” if it was; and the variable “number of cross-posts” (*H10*) is discrete.

To operationalize our time-control variables, we proceed as follows: Reddit submissions are timestamped in the UTC format when retrieved from the Pushshift submission dumps, but we recode submissions with our time-controlling categorical variables “weekday/weekend” and “time of day” using U.S. Pacific time. We do so for two reasons: a disproportionately high number of Reddit users live in a U.S. time zone [84]; and several violent BLM events happened on the West Coast, especially in Portland, Oregon, marking it an important area for (violent) BLM protest [85]. For the variable “weekday/weekend,” we classify Saturday and Sunday as “weekend” and all other days of the week as “weekday.” We code the variable “time of day” as “morning” for submissions posted between 4am and 12pm, as “afternoon/evening” for those posted between 12pm and 8pm, and as “night” for those posted between 8pm and 4am.

For coding our other control variable, “type of news outlet,” we also use information retrieved from the website mediabiasfactcheck.com and apply the categorical labels “newspaper,” “magazine” (merging “magazine” and “journal”), “news agency,” “organization/foundation,” “radio station” (merging “radio” and “radio station”), “TV station” (merging “TV station” and “TV station/website”), and “website” (merging “website,” “app/website,” “website/newspaper,” and “website/video”). In our analysis, the latter one is considered to be digital-born media, and the others are considered to be digital extensions of traditional media. (For more information regarding the variable distributions, please see Supporting Information, Descriptive overview of variables.)

### Data collection and VGG19 image classifier

#### Social media platform Reddit

Although traditional media still serve as important news sources, consuming news through social media websites has become increasingly important as sources of news [5,86], even beginning to replace traditional media [87]. Thus, analyzing the relationship between digital news consumption and attention distributions on social media websites is essential for understanding one integral part of today’s hybrid media system [7,8]. In our analysis, we focus on Reddit because it is often regarded as “the front page of the Internet” [88] and is one of the most popular social media news sources [5], known as a place to share new social trends, political opinions, and innovative ideas [89]. Moreover, Reddit has a unique structure compared to other social media platforms. It is divided into various subreddits in which “users actively choose to participate in specific discussion groups that interest them … rather than creating friend networks” [89]. Thus, “social connections are less salient on Reddit, which seems more centered on the content” [67], making the platform a particularly good choice as the foundation for our analysis of the discourse on and attention paid to the BLM movement.

When evaluating Reddit’s communication and interaction landscape, it is critical to remember that its audience of users is skewed in certain ways that could affect results; for example, the social composition is biased toward American men of middle age with left-leaning political views [84]. To account for these biases to a degree we deem possible, we analyze three relatively politically neutral subreddits that are highly popular for sharing news on Reddit and are commonly used as subreddits of analysis in this context [see, e.g., 90]. Moreover, this study aims to analyze the effect of mainstream news reporting on user attention to capture the general relationship between shared news and user attention. Therefore, we intentionally focus on the most prominent subreddits for sharing news with strict community rules, mainly featuring mainstream media outlets [91]. We disregard more radical or fringe news subreddits on Reddit as they attract a different audience, operate according to different community rules, share different types of content, and, therefore, contain a different logic of attention distribution [92].

The first two subreddits are r/worldnews, with 28.6 million users (as of April 16, 2022), and r/news, with 24.5 million subscribers; both are premier subreddits for exchanging and discussing international news. We also include the subreddit r/politics, with 8 million members; it is dedicated exclusively to U.S.-specific news. These three subreddits have similar community rules and standards for user submissions (regarding title length, title content, and timeliness of news articles; see submission guidelines of each subreddit). Submissions posted in all three subreddits consist of a link to a news article and a title matching the title of the news article, which allows for a direct comparison; only one, r/politics, allows the posting of thumbnails. Since all three subreddits are dedicated to sharing news articles only, a similar type of image attached to a submission’s linked article can be expected. This, in turn, enhances the comparability of all three subreddits and the submissions to them and paves the way for a correct categorization of the image classifier, which is also trained on protest-related news images.

#### Reddit BLM dataset

We use the monthly Pushshift submission dumps [88] to gather the data of the three subreddits, which is regularly done for collecting Reddit data [e.g., 93]. We downloaded the Pushshift submission dumps for May 2020 to May 2021 (105GB) on May 9, 2023, and filtered the data regarding the three subreddits of interest r/worldnews, r/politics, and r/news (830MB), which led to 1,887,202 submissions. With our objective of evaluating the BLM movement reignited in 2020, we reduced the data toward the period of analysis from May 25, 2020, the day of George Floyd’s murder, to May 25, 2021. This yielded 1,763,659 submissions. We removed all submissions whose titles did not match one of the three keywords, “black lives matter,” “george floyd,” or “blm,” leaving a total of 17,083 submissions relevant to our analysis.

Note that we also tested how an increased keyword set related to our analysis topic would increase our data corpus. We, therefore, added the BLM-specific keywords “police violence,” “breonna taylor,” and “rayshard brooks” to our keyword set; this, however, did not result in a significant increase in submissions and added more noise to the dataset (for more information, see Supporting Information, Data availability statement). We thus decided to continue working with the initial keyword set.

For our data cleaning process, we removed a particular type of submission from our dataset, so-called “megathreads,” which summarizes a multitude of submissions about highly popular events and typically receives a disproportionate amount of attention; this reduced the number of submissions to 17,069. After dropping all missings regarding our control variables from mediabiasfactcheck.com (“political leaning” (*H4*), “factual reporting” (*H5*), “traffic” (*H6*), and “type of news outlet”), we received 11,813 submissions.

Given our goal to analyze the impact of images displaying violence, the next step is to collect the images from the news articles referenced in our dataset’s submissions. We use the images from the linked news outlets because the three subreddits allow only submissions with news titles and news article links, thus setting the focus on the originating news outlet and its images. We assume that users who engage with the submission also click on the URL link, as it is the primary purpose of the submission itself. We use the Python library “Newspaper3k” to extract the main images (also used as thumbnails in r/politics) from each news article in our dataset. The “main image” of an article is defined within the source code of each website and typically corresponds to the first image on the website. Note that if Newspaper3k could not extract an image due to, for example, captchas of a news outlet’s website, we manually extracted the main image in the cases possible. To test how well Newspaper3k extracted the correct main image of a news article, we manually evaluated 200 random images retrieved by Newspaper3k. Of 200 images, 152 were correctly detected as the main image (76.0%). As nearly all other images that were not labeled as the main image were still part of the news article and most often the second or third image on the website, we deemed this result acceptable and continued working with those images. After downloading the images detected by Newspaper3k and manually added by us, we retrieved 9,373 images for our dataset (no image was available for 2,440 submissions).

For our analysis, we are interested in submissions that received at least a minimum of attention and thus have been recognized by Reddit users. We, therefore, removed all submissions that received zero comments which reduced the dataset to 5,876 submissions (yet, to sustain our results, we also ran a negative binomial regression model that included submissions with zero comments, yielding similar results for the main effects; for more information, see Supporting Information, Negative binomial regression models). After dropping all missing values of the other variables related to our analysis, our final dataset consists of 5,873 observations.

### Training dataset for image classifier

Prelabeled training and validation datasets are necessary to build an automated image classifier that can label an image as violent (= 1) or nonviolent (= 0). Therefore, we use images from the “UCLA Protest Image Dataset” of Won et al. [94], a prelabeled protest-related dataset of 40,764 images. In this dataset, “violence” is operationalized as a continuous variable; we transfer that labeling to our dichotomous coding scheme by manually applying Rucht’s [68] violence definition to the images. Using their dataset’s most- and least-violent images, we end up with 2,566 images in the training dataset and 700 in the test dataset. Moreover, to train our image classifier with general protest images *and* BLM-specific images, we automatically download images via the search engine “Bing,” employing 25 BLM and protest-related related keywords in English and German (for the entire keyword set, see Supporting Information, Data availability statement). We classify them following the same procedure outlined above. Finally, we enlarge our dataset using image augmentation, resulting in 7,972 violent images and 8,986 nonviolent images in the training dataset and 2,854 violent images and 4,088 nonviolent images in the validation dataset (for more information related to the dataset creation, please see Supporting Information, Data availability statement).

### Transfer learning & performance of VGG19 image classifier

We use a convolutional neural network architecture to classify violent images for two reasons: the computational power and memory space of CNNs are relatively inexpensive due to parameter sharing; and CNNs autonomously select important image features based on automatized feature extraction and without human supervision, thus achieving state-of-the-art results while avoiding human biases [95] (see Supporting Information, Image classifiers, for different models that we tested but that performed worse than CNNs).

Research has repeatedly shown that using a CNN pre-trained on general image features increases the model performance once finetuned to the specific classification task through transfer learning [see, e.g., 96]. We follow this advice and apply the three state-of-the-art CNN architectures VGG19, ResNet50, and EfficientNetB4 [97,98] pertained on the ImageNet-1k dataset [99] from the Python deep learning libraries “TensorFlow” and “Keras.” In addition, we create our own CNN architecture as a fourth model for comparison. As the VGG19 model performed best after finetuning and running the different models, we continue utilizing this CNN architecture for our analysis. In the following, we go into more detail regarding the training and performance of the VGG19 model (for more information regarding the architecture, training, and evaluation of the other models, please see Supporting Information, Image classifiers).

We use transfer learning to adjust the VGG19 model to our classification context of classifying images as violent (1) or nonviolent (0). Therefore, we freeze all layers except for the last fully connected layer of the pre-trained model, as unfreezing more layers does not yield better training results. We replace the last fully connected layer with a dense layer that applies a sigmoid function as an activation, which returns a number between 0 and 1 and is, therefore, suitable for our binary image classification. We compile the model with the adam optimizer, a learning rate of 0.001, and a binary cross-entropy (BCE) loss function, resulting in a binary classification corresponding to our classification of “violent” and “nonviolent.” We split the training data into 70% training images and 30% validation images. We also implement an early stop callback function with a minimum delta of 0.1% and a patience parameter of 10 within our training process to prevent overfitting. Thus, our training will stop if our validation loss does not improve within ten epochs by at least 0.1% compared to its best value. We start training the model with 40 epochs and a batch size of 32. As the model’s validation loss continuously improves by at least 0.1%, the training ends after 40 epochs.

Our VGG19 image classifier achieves an accuracy value of 91.65% on the training data and 90.29% on the validation data. When assessing the performance on the test dataset, the classifier achieves an accuracy of 91.84%, which is comparable to the training and validation accuracy. The classifier achieves a precision score of 87.78%, a recall score of 93.13%, and an F1 score of 90.37% on the test dataset. Moreover, when assessing the performance of our BCE loss function, both the training and validation loss continuously decreased, which is a sign that the model picks up essential image features without overfitting the data (in epoch 40, a loss of 0.22 on our training data and 0.24 on our validation data). Thus, the classifier’s performance is sound and allows for a valid image classification (for more information on the VGG19 model performance, please see Supporting Information, Image classifiers).

## Results

As a first step, we classify our BLM images with our VGG19 image classifier, resulting in 5,486 images classified as “nonviolent” (93.4%) and 387 as “violent” (6.6%). To validate whether our image classifier performs as well on our BLM dataset as on the training, validation, and testing set, we manually label 200 random BLM images and compare our classification result with the classifier’s results. Of 200 images, 180 are correctly labeled (90.0%), which aligns with the previous validation metrics and therefore supports the classifier’s performance in our BLM protest context. Moreover, when calculating the sentiment of a submission’s title with the BERT model, we receive 1,253 (21.3%) positively connoted titles and 4,620 (78.7%) negatively connotated titles. To validate these classification results, we draw a random sample of 200 submission titles and manually label them as “positive” or “negative.” The manual and automatic sentiment classification matches in 168 out of 200 cases (84.0%). This value is in line with the previous validation and test accuracy of the BERT model, supporting the model’s sound performance on our Reddit BLM data. We thus have all the necessary data to run our data analysis (for a description of all variables, see Supporting Information, Descriptive overview of variables).

The count nature of our dependent variable, “number of comments,” makes a Poisson or negative binomial regression model most suitable for the analysis. To test whether a Poisson or negative binomial regression would be more appropriate for our data, we conduct a likelihood ratio test for overdispersion in count data (“odTest” function from the R package “pscl”). The likelihood ratio test estimates whether the data’s variance equals its mean, which is the assumption of a Poisson model. The test results show that our model’s data’s variance is greater than the mean (which is also supported by the positively skewed data distribution of our dependent variable “number of comments”), implying a significant data overdispersion. Thus, a negative binomial regression, which relaxes the assumption regarding variance equaling mean, is a more suitable model choice for our data [for more information, see 100] (for more information regarding our model choice, please see Supporting Information, Negative binomial regression models).

To run the negative binomial regression, we use the “glm.nb” function from the R package “MASS.” Note that we did not standardize the variables before running the models, as we did not include any interaction terms and have only one numeric control variable, “number of cross-posts.” Likewise, standardizing the variables by subtracting the mean and dividing them by the standard deviation before running a negative binominal regression impedes the interpretability of the results. Thus, it is often omitted when performing this type of analysis [for more information, see, e.g., 101]. To further check the suitability of our model, we control for multicollinearity within our model through the variance inflation factor (VIF). Doing so, we detect no worrisome correlation between our variables (all values of VIF < 5, with the variables “type of news outlet” displaying the highest values of 1.8). Moreover, we check for outliers in our data which could bias the results. Therefore, we apply Cook’s distance but detect no data point that surpasses the critical value of 1 [102]. Thus, our model is suitable for our analysis by providing meaningful results.

Table 2 shows the results of our negative binomial regression analysis (to test the robustness of our model results, we ran different negative binomial regression models based on variations of the dataset and variables; when comparing the results, only small deviations from our results can be detected, sustaining our findings; for more information, see Supporting Information, Negative binomial regression models).

**Table 2:** Negative binomial regression model (standard errors in parentheses, p values in square brackets).

	dependent count variable: number of comments
violent image	-0.012 (0.072) [0.866]
BERT sentiment negative	0.132** (0.044) [0.003]
political leaning conservative	-0.211** (0.073) [0.004]
political leaning conspiracy	-0.215 (0.990) [0.828]
political leaning liberal	-0.099• (0.058) [0.087]
factual reporting high	0.083* (0.042) [0.046]
factual reporting low	-1.005*** (0.124) [0.000]
traffic high	-0.246*** (0.066) [0.000]
traffic minimal	-1.198*** (0.218) [0.000]
subreddit news	0.977*** (0.048) [0.000]
subreddit worldnews	0.764*** (0.069) [0.000]
NSFW	-1.264 (0.804) [0.116]
link flair	-0.566*** (0.037) [0.000]
number of cross-posts	0.896*** (0.014) [0.000]
weekend US pacific	0.249*** (0.042) [0.000]
morning US pacific	-0.005 (0.039) [0.898]
night US pacific	-0.097• (0.050) [0.054]
type of news outlet magazine	0.263** (0.086) [0.002]
type of news outlet news agency	-0.766*** (0.102) [0.000]
type of news outlet organization/foundation	-0.123 (0.182) [0.501]
type of news outlet radio	-0.375* (0.154) [0.015]
type of news outlet TV station	0.052 (0.047) [0.266]
type of news outlet website	0.096• (0.064) [0.064]
Constant	3.276*** (0.093) [0.000]
Observations Null deviance Res. deviance AIC Theta Std. Error	5,873 14,121 on 5,872 df 6,903 on 5,849 df 50,416 0.557 0.009

Note: •p<0.1; *p<0.05; **p<0.01, ***p<0.001.

Note that the following interpretation of the individual coefficients assumes that all other variables are held constant. Also, the nature of social media data only allows for correlative associations between the variables and cannot be causal. Thus, the following result will be interpreted against this background.

When evaluating the coefficient of our main independent variable, “violent image” (-0.012), we find–contrary to our assumption–that images featuring violence are negatively associated with the number of comments, yet not on a statistically significant basis (*H1*). Therefore, this finding cannot be generalized. When analyzing the results of attributes related to the news item, the coefficient of our title’s sentiment analysis with BERT turns out to be significant (*H2*), with a p-value of 0.01, demonstrating that negative titles increase the logged count of number of comments by 0.132. Thus, our results show that a submission’s title not only has a significant (see p value) but also a greater (see coefficient) effect on user attention compared to images (*H3*).

When evaluating the effects of attributes related to the news provider, conservative and liberal news outlets significantly negatively impact our dependent variable compared to neutral news outlets (p = 0.01 and p = 0.1, respectively, coefficient of -0.211 and -0.099, respectively) (*H4*). Both high and low factual reporting of a news outlet significantly impact the dependent variable compared to mixed factual reporting (*H5*). High factual-reporting news outlets positively affect the logged count of number of comments by 0.083 compared to mixed factual-reporting outlets. In contrast, low factual-reporting news outlets are negatively correlated with a coefficient of -1.005 (p value = 0.05 and 0.001, respectively) compared to mixed factual reporting outlets. Analyzing the effects of a news website’s traffic (*H6*), both news outlets with high and minimal traffic are negatively associated with the logged count of number of comments compared to news outlets with medium traffic (both with a p value of 0.001 and a coefficient of -0.246 and -1.198, respectively).

When examining the variables related to Reddit-specific affordances and community rules, the type of subreddit (*H7*) plays a significant role. Both r/news and r/worldnews positively correlate with the logged count of number of comments compared to r/politics (both a p value of 0.001 and a coefficient of 0.977 and 0.764, respectively. Whether a submission is NSFW (*H8*) does not substantially impact the dependent variable. Yet, link flair (*H9*) and the number of cross-posts (*H10*) turn out to be highly significant, with a p-value of 0.001. If a submission is tagged with a link flair, it negatively correlates with the logged count of number of comments by -0.566, whereas the number of cross-posts is positively associated with the dependent variable with 0.896.

Finally, when evaluating the effects of our control variables, it turns out that if a submission is posted on a weekend compared to a weekday, the logged count number of comments is positively affected by 0.249 (p value = 0.001). The timing of posting a submission also shows a significant effect. Submissions published at night negatively impact the dependent variable by -0.097 (p value of 0.1) compared to submissions posted during the afternoon/evening (note that for UTC, U.S. Central, U.S. Eastern times, and Central European times, we receive the same effects for the weekend). When examining the effect of the type of news outlet, digital-born media types–namely websites–and digital extensions of traditional media, namely, magazines, news agencies, and radio stations–have a significant effect compared to the reference category newspaper (which belongs to the category digital extension of traditional media outlets). Websites have a significant positive association with 0.096 and a p value of 0.1. Submissions that feature links to news agencies or radio stations significantly negatively correlate with the dependent variable (coefficient of -0.766 and -0.375, respectively, and a p value of 0.001 and 0.05, respectively). In contrast, submissions featuring links to magazines have a positive impact of 0.263 and a p value of 0.01 compared to newspapers (note that newspaper was chosen as a reference category because it has the greatest number of observations).

When comparing the AIC (Akaike Information Criterion) value of our model with those of the other control models (see Supporting Information, Negative binomial regression models), our model is either as suitable or far more suitable for representing the data (AIC value is considerably smaller compared to two models and nearly as big as the AIC of another model). Thus, our model fits the data well compared to the other models, making it a reliable choice for analyzing our data. Also, as the residual deviance is significantly smaller than the null deviance, we can infer that our model explains a significant amount of the variation in the data compared to the null model.

## Discussion

Based on an evaluation of our empirical results, we can summarize the answer to our research questions as follows: violent images *do not* affect user attention on Reddit, whereas other attributes related to news reporting–such as the political leanings and factual reporting of a news outlet, a news outlet’s title, Reddit community rules and subreddits, the timing of a submission, and a news outlet’s media type–are *significantly* correlated. In this section, we discuss these results in the context of our hypotheses by providing theoretical and practical implications.

In light of our empirical findings, we cannot verify that a violent visual representation leads to greater user attention and must therefore reject *H1*. Our findings align with previous studies that could not detect a significant relationship between online images featuring violence and attention in a protest context [16,17]. It may be that violent images depict a form of negativity that is too intensive for social media users who prefer less negativity in their news consumption [3] or prefer shallow social content for entertainment purposes [66]. Thus, our results confirm that image virality on social media–such as the images of cats that enjoy such tremendous popularity [67]– is highly context-sensitive.

In addition, we need to keep in mind that in our current “attention economy” [103], there is an abundance of social media content competing for user attention, which might diminish the effect of images featuring violence. Thus, our analysis shows that it is difficult to replicate existing findings from traditional media research regarding the effects of news featuring violence on attention to the online world. Although previous research has shown that offline news consumption habits can be transferred online [60], one must remember that previous research focused on the type of news outlet readers prefer. Thus, our findings should not be seen as a contradiction to earlier findings but rather as a supplement focusing on the type of reporting.

Moreover, one must note that news consumption on social media follows an entirely different logic than the linear news exposure of traditional media [104]. This could partially explain the divergent effects of news featuring violence in different media settings. Future research could expand these insights by combining quantitative and qualitative approaches, as proposed by Mitchelstein and Boczkowski [61].

Beyond these theoretical explanations for our non-confirmative findings, we also need to consider some empirical research design decisions that may have affected our results. First, the operationalization of “violence” according to Rucht’s [68] definition is quite strict. When manually assessing the BLM image data, we find that several images could be identified as “violent” were we to adopt a reasonably strict but more inclusive definition.

Second, a binary operationalization is prone to classification errors. The portrayal of violence in images is a nuanced and complex concept and operationalizing it by utilizing a continuous variable would preserve additional information, which might improve our results.

Third, we analyzed the main images used in the news articles submitted to Reddit. While we consider this a reasonable choice, essentially restricting our analysis to the strongest available signal for the visual portrayal of violence in images, extending our research to additional visual depictions of violence on websites linked in submissions would be interesting. Likewise, the subreddits utilized for our analysis prohibit directly posting images within submissions. As these subreddits are the most prominent for news sharing and, thus, are important for analyzing user attention in relation to news articles, we consider this choice appropriate. Yet, based on the subreddits’ restrictions on directly posting images, we are likely underestimating the effect of (violent) images. We invite future research to extend our analysis by including subreddits related to the sharing of news that allow for the direct posting of images.

Finally, the operationalization of user attention could be extended by, for example, evaluating the content of comments made to the analyzed submissions. That would allow for the analysis of interaction dynamics triggered by violent content.

While we do not find a significant effect of the portrayal of violence in images, several of our other news-related attributes show promising results. Foremost among these, we find when analyzing attributes related to the news item that negative titles correlate significantly with the attention paid to a submission on Reddit in the context of our study. Hence, we can confirm *H2* and the implications of the negativity bias hold for the relationship between text with a negative sentiment and user attention. This finding aligns with previous results on the positive relationship between negative emotional language and information spread online [3,105]. In addition, it emphasizes the importance of “negativity” when reporting news [64]. Yet, when evaluating these findings, the internal structure of the considered subreddits needs to be taken into account. According to the rules of the subreddits we analyzed, a valid submission features a title and a link to a news outlet’s website. Submissions not following these rules are removed immediately, indicating that Reddit and its communities mediate and restrict the information exchanged. These moderating rules emphasize a submission’s title, potentially enhancing its effect on user attention. This finding again highlights the importance of considering the logic of a subreddit’s affordances and community rules to analyze user attention [69].

In contrast to our assumptions, we cannot confirm that images significantly impact user attention more than written text. Considering that our study shows that a submission title’s negative sentiment has a greater coefficient and more significant effect on user attention than violence featured in the accompanying images, the picture superiority effect does not hold in our analysis. Thus, we must reject *H3*. Other studies have come to similar conclusions that although news shared on a “news aggregator website” featuring images positively affects the readership, images negatively affect the number of comments an article receives due to their distracting character [106]. Since Reddit is a social media website known for encouraging the posting of comments and the discussion amongst users [107], it operates entirely differently than news aggregator websites. Thus, we consider our results as complementary to previous research. As an extension of our work, future research could analyze subreddits with diverging community guidelines, which could impact the prominence of images and, thus, the attention paid to them.

We observe several interesting effects when analyzing the impact on user attention of attributes related to the news provider. In contrast to previous research that found no relationship, in the context of negative news, between a news outlet’s political stance and user engagement [105], we are able to show that conservative and liberal news outlets correlate with user attention negatively compared to neutral reporting–contrary to our assumptions. Thus, we must reject *H4*. Therefore, our findings indicate that users interested in BLM favor a politically neutral reporting style when informing themselves about news posted in the three subreddits of analysis. Since a tense political climate surrounds BLM [29], one reason for this finding could be that users prefer their information consumption to be less politically charged.

Unlike previous research [75], we did find a significant positive correlation between high factual reporting (“true news”) and a negative correlation between low factual reporting (“false news”) and user attention compared to mixed factual reporting. Consequently, we must reject *H5*. One possible explanation is that previous research focused on the relationship between the rapid spread of tweets featuring misinformation and user attention [75], whereas we focus on the effects on user attention of a digital news outlet’s reporting style. Thus, it seems that the audience for news about BLM is more interested in factual reporting, which could be attributed to the topic’s political relevance and the desire to counter existing polarized positions [29]. This finding also aligns with the impression that users interested in BLM on Reddit favor less politically charged news content. Future research could build upon these initial findings and dive deeper into reporting’s level of factuality and attention dynamics on social media.

In *H6*, we assumed a positive relationship between the popularity of a news website and the attention received by submissions to it. Our data, however, reveal a negative correlation between very popular and very unpopular news websites with user attention. Therefore, we reject *H6*. This finding can be seen as in line with the findings regarding factual reporting and political leaning: When looking into our data, popular news outlets often feature a low- to mixed factual reporting style and tend to be politically charged (e.g., Fox News, Daily Mail, or The Guardian). Thus, we observe a tendency that users prefer medium popular outlets that are visible (not minimal traffic), with factual and politically unbiased reporting (e.g., Bring Me The News or KMSP Fox 9).

The effects of our attributes related to Reddit-specific variables also lead to interesting findings that highlight the importance of a platform’s affordances and community moderating rules. While we cannot confirm that the attachment of a NSFW tag (*H8*) significantly correlates with user attention, we see that a subreddit in which a submission is posted (*H7*), the flair tag (*H9*) attached to a submission, and the number of cross-postings (*H10*) does correlate with user attention. Based on these findings, we again cannot confirm that more violence (NSFW) leads to greater user attention. We refrain, though, from overstating the significance of this finding due to the variable’s highly skewed distribution (see Supporting Information, Descriptive overview of variables).

Likewise, the picture superiority effect also does not hold for the posting of thumbnails (r/politics), which highlights again the prominence of a submission’s title and the particular structure of the subreddits we considered. It is interesting to observe that r/news and r/worldnews correlate positively with user attention compared to r/politics. This could be a sign that users interested in BLM are rather looking for an international perspective on BLM (r/news, r/worldnews) as it is a worldwide protest [21] compared to solely reading BLM news related to the US context (r/politics).

Finally, a subreddit’s community rules and moderation (link flair) significantly affect whether a post will be visible to Reddit users and thus receive attention. The positive relationship between cross-posts and user attention also confirms this effect of visibility on user attention. Therefore, understanding Reddit’s specific affordances and community rules (which differ highly between the multitude of subreddits hosted on Reddit) is important for capturing attention distributions on the platform. Summarizing these findings, we must reject *H7* and *H8*, but we confirm *H9* and *H10*.

When evaluating the effects of our time controls, “weekend” and “time of day” turn out to be significant. Our results show that Reddit users are more active during the weekend and the afternoon/evening, resulting in higher attention paid to submissions posted on the weekend and during that period (12pm to 8pm) than those posted at night. The first finding is unsurprising, as users tend to have more time on weekends to engage with social media than on weekdays. In contrast, it is somewhat surprising that users tend to consume and comment on news more often during the day compared to the night (negative effect of submissions posted during the night). Yet, this finding aligns with our impression that users of the subreddits we analyzed who are interested in BLM prefer to consume their news in an organized fashion (significance of regular working hours and not at night). However, these results based on U.S. time zones must be treated with caution since BLM is an international movement with an audience that likely engages with BLM news content from different time zones across the globe. Nevertheless, these U.S.-centered findings are still interesting to take into account, especially given that most Reddit users are U.S.-based and the BLM movement originates from the United States.

Regarding the type of news outlet, we also find significant effects on user attention. In our analysis, websites considered digital-born news outlets and magazines, news agencies, and radio stations considered digital extensions of traditional media significantly correlate with user attention compared to newspapers. We can observe that easy-to-read news outlets such as magazines (e.g., *Newsweek*, *Rolling Stone*) and specific websites (e.g., Buzzfeed News) are positively associated with user attention compared to newspapers. Moreover, news agencies such as Reuters are negatively correlated with user attention, which is not surprising given that the primary role of news agencies is to distribute news to their customers compared to publishing news themselves. Likewise, users seem to prefer written content compared to audio content (negative association with radio stations). These findings indicate that the types of news outlets users favor when informing themselves about BLM on Reddit should be factual but not too cumbersome to read. Users have a positive relationship with digital-born news outlets, whereas they have mixed feelings regarding the digital extension of traditional media, as news outlets correlate positively and negatively with user attention.

We want to stress that these findings relate solely to the three subreddits analyzed within this study and are, therefore, not generalizable to other subreddits or social media platforms. Reddit’s subdivision into different subreddits makes its structure highly different than other social media platforms such as Instagram and TikTok. Thus, one must keep in mind that each subreddit and social media platform is differently structured, operates according to different community rules, and entails divergent platform affordances [108], which impacts the salience of different news topics shared and the user engagement and attention paid to them [109].

## Conclusion and limitations

Our work contributes to research on the dynamics of user attention on social media in the context of digital news reporting on the BLM movement on Reddit. This work is a first important step for improving our general understanding of attention dynamics on social media in the context of digital news reporting, especially violent news reporting, about social movements, which in turn can influence the perception of and support for such a movement [22–24,26]. Since the consumption of news on social media platforms has become more important than ever before [3–5], this analysis provides valuable insights into a growing news market that is an essential part of the hybrid media system [7,8].

Our six key takeaways are:

- Findings from traditional media research cannot be directly replicated within the online world. Although research suggests that offline and online news consumptions often follow similar patterns [60,61], one must take into account that both environments follow different logics [104]. On social media, in particular, an abundance of content competes for user attention [103], which differs from the linear news exposure in traditional media.
- In our analysis, it is not a singular effect (violence in images) but the interplay of several attributes related to the news item, news provider, and community rules and platform affordances that are essential for capturing attention distributions on Reddit. This highlights the complexity of the social media landscape.
- Submission titles with negative connotations correlate positively with user attention compared to titles with positive sentiment. Thus, we find indications for a negativity bias with respect to written content.
- The picture superiority effect cannot be confirmed in our analysis, given that a submission’s headline has a greater coefficient and a significant impact on user attention compared to submissions featuring violent images. In our discussion, we point to several potential explanations for this finding.
- Users consuming BLM-related news tend to exhibit special characteristics by preferring negatively connotated yet politically non-biased, factual, and easy-to-read news published during the day and on weekends. Thus, taking a news topic’s audience into account is highly important for understanding the popularity of news content.
- Platform-inherent affordances and community rules make essential contributions to the understanding of user attention dynamics, demonstrating that “both crowds and systems work together to make decisions on what information is important” [3].

These findings emphasize the need to expand our understanding of news consumption and attention distributions in an increasingly digitalized world since “an operational and useful democracy relies on an educated and well-informed population” [110]. This paper contributes another element to this field of research and paves the way for potentially exploring broader questions, such as whether the detected effects of this paper also hold for the digital news coverage of other social movements on other social media platforms.

While the findings presented in this study offer important theoretical and practical insights, they also have to be seen in the light of their limitations—which open up several promising avenues for future research. First, it would be interesting to measure the dynamics of user attention in a news context over time. Doing so would allow for evaluating social media’s effect on digital news consumption dynamics by revealing what kind of news attracts the greatest user attention and when.

Second, while we deem our choice of subreddits as a justifiable first step for analyzing the relationship between news and user attention since they represent very important subreddits for sharing news on Reddit, they are not free of biases that affect our study. As the three subreddits do not allow for the direct posting of images, we recognize that we are underestimating the effect of (violent) images on user attention. Future research is invited to add further news-related subreddits with less strict community rules allowing for the direct posting of images. Further, while we intentionally focused on mainstream news outlets in our analysis to capture the general relationship between shared news and user attention, it would be interesting to expand our analysis to more radical subreddits with different community rules, audiences, and content compared to mainstream subreddits, including those that encourage the sharing of conspirational and fringe news. This extension would provide additional and interesting insights regarding the relationship between news and user attention on Reddit. Beyond including more and diverse subreddits, adding other social media platforms and data about different social movements, as well as including additional explanatory attributes for user attention, would extend the scope of this research and add to its generalizability.

Third, analyzing the comment structure and user-generated content in response to a submission could be a welcome addition to investigate user attention further. Focusing on interaction dynamics between users in the context of content featuring violence could deepen our understanding of user reactions to violence portrayed online.

## Supporting information

S1 Fig Histogram of monthly distribution of Reddit BLM submissions. (TIF)

S2 Fig Histogram of dependent variable number of comments. (TIF)

S3 Fig Training and validation accuracy of BERT model. (TIF)

S4 Fig Accuracy of CNN models. (TIF)

S5 Fig Accuracy of VIT models. (TIF)

S6 Fig VGG19 model loss function. (TIF)

S7 Fig VGG19 model precision, recall, and F1-Score. (TIF)

S8 Fig VGG19 model receiver operating characteristic (ROC) curve. (TIF)

S9 Fig VGG19 model confusion matrix of test dataset relative values. (TIF)

S10 Fig VGG19 model confusion matrix of test dataset absolute values. (TIF)

S1 Table Descriptive overview of variables. (DOCX)

S2 Table Own convolutional neural network architecture. (DOCX)

S3 Table Negative binomial regression results of models with different datasets and variables (standard error in parentheses, p values in square brackets). (DOCX)

S1 File Appendix–Attention-grabbing news coverage: Violent images of the Black Lives Matter movement and how they attract user attention on Reddit. (DOCX)

S2 File (ZIP)
