# Supplementary figures and images for: Attention-grabbing news coverage: Violent images of the Black Lives Matter movement and how they attract user attention on Reddit
Source: PLoS One. 2023 Aug 9;18(8):e0288962. doi: 10.1371/journal.pone.0288962 (PMC10411814; doi:10.1371/journal.pone.0288962)

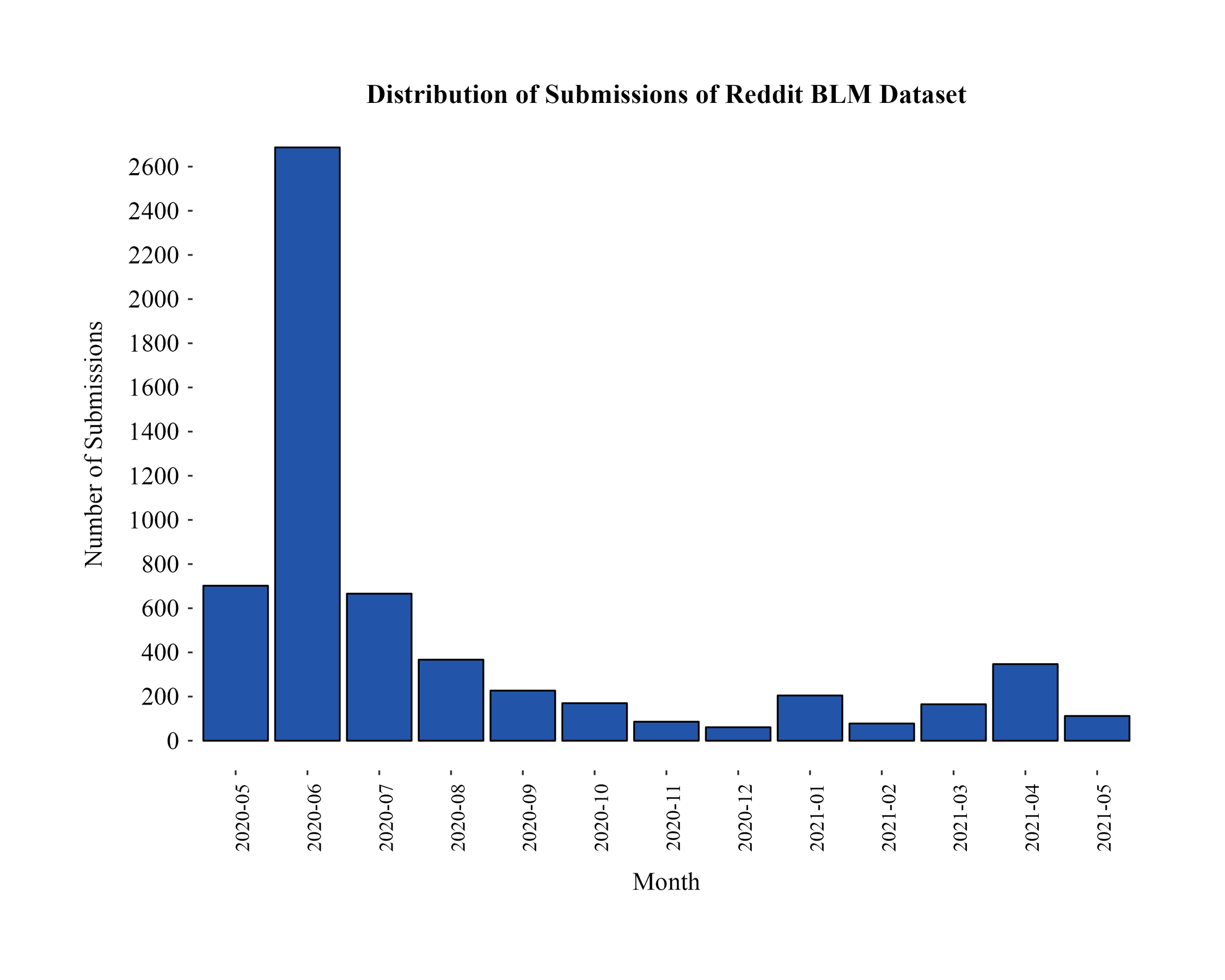

Supplement: S1 Fig — (TIF) [file pone.0288962.s001.tif]

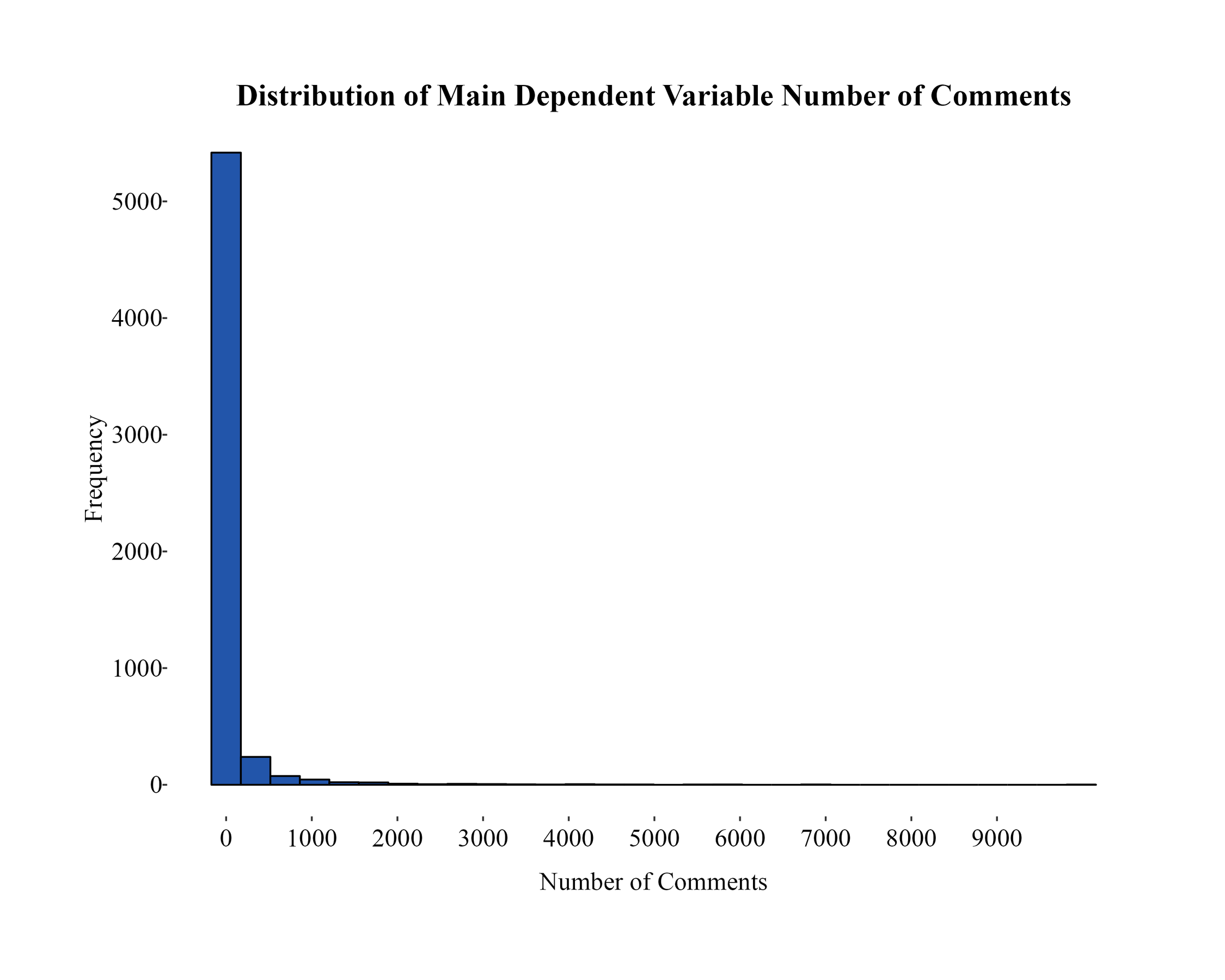

Supplement: S2 Fig — (TIF) [file pone.0288962.s002.tif]

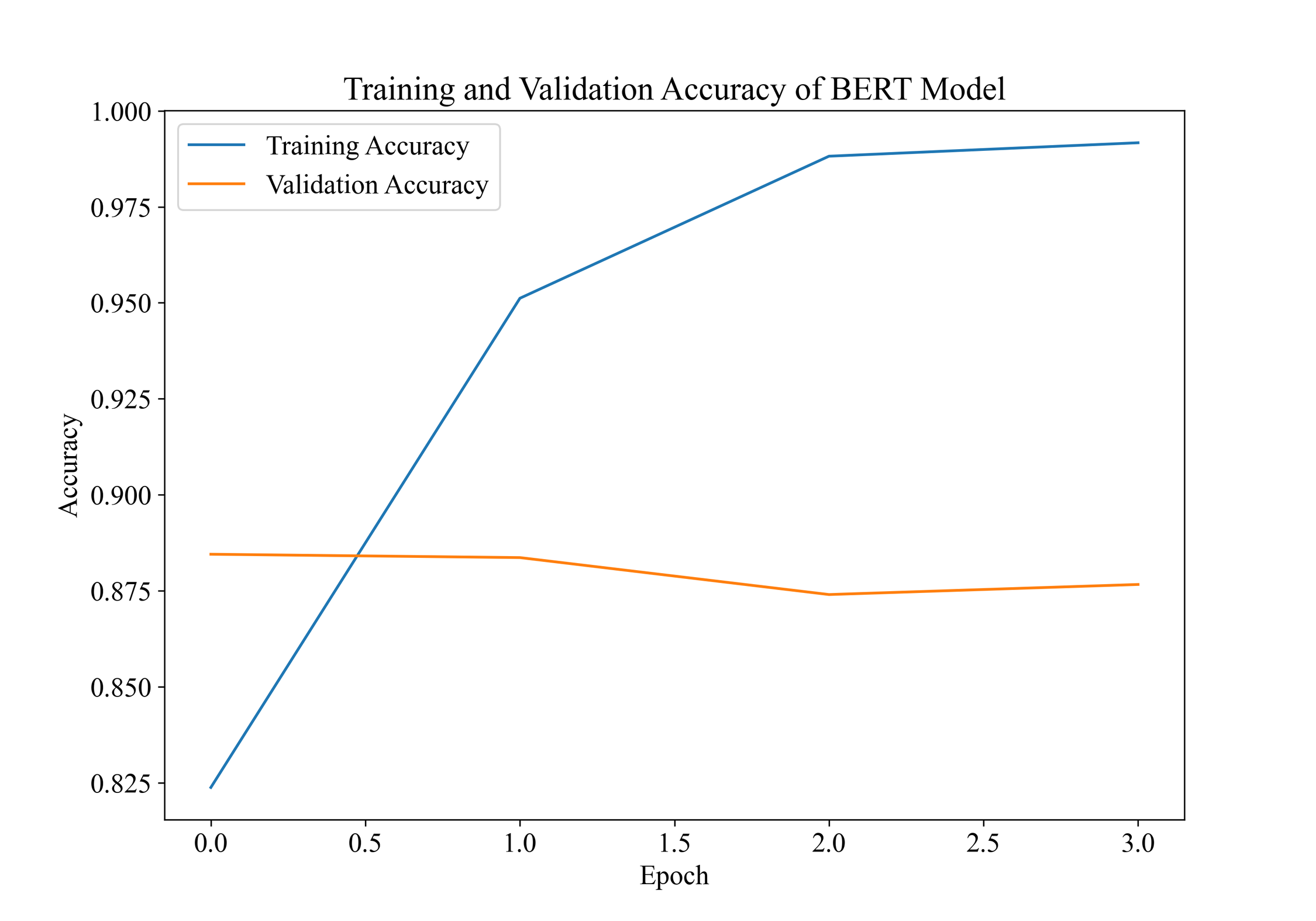

Supplement: S3 Fig — (TIF) [file pone.0288962.s003.tif]

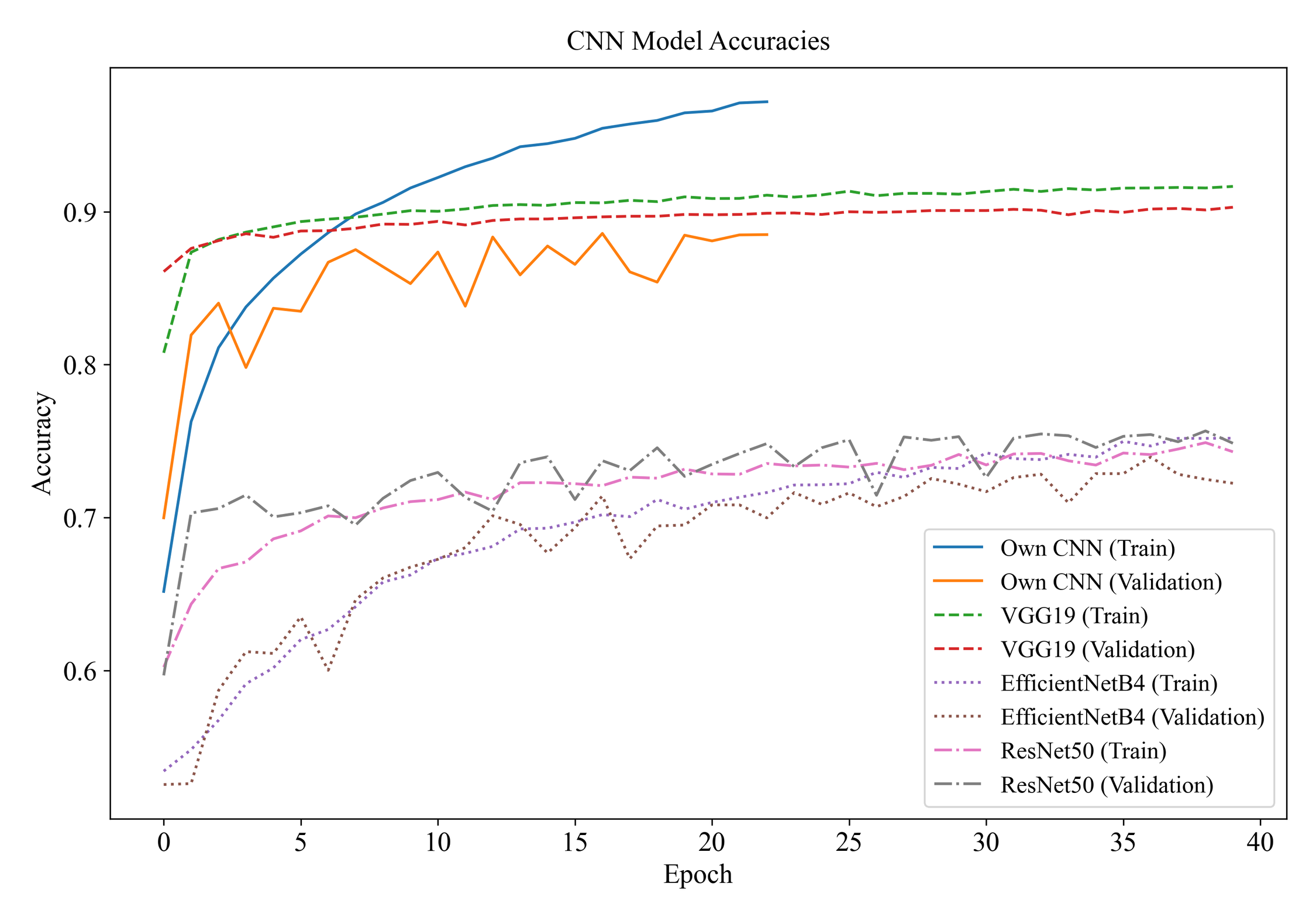

Supplement: S4 Fig — (TIF) [file pone.0288962.s004.tif]

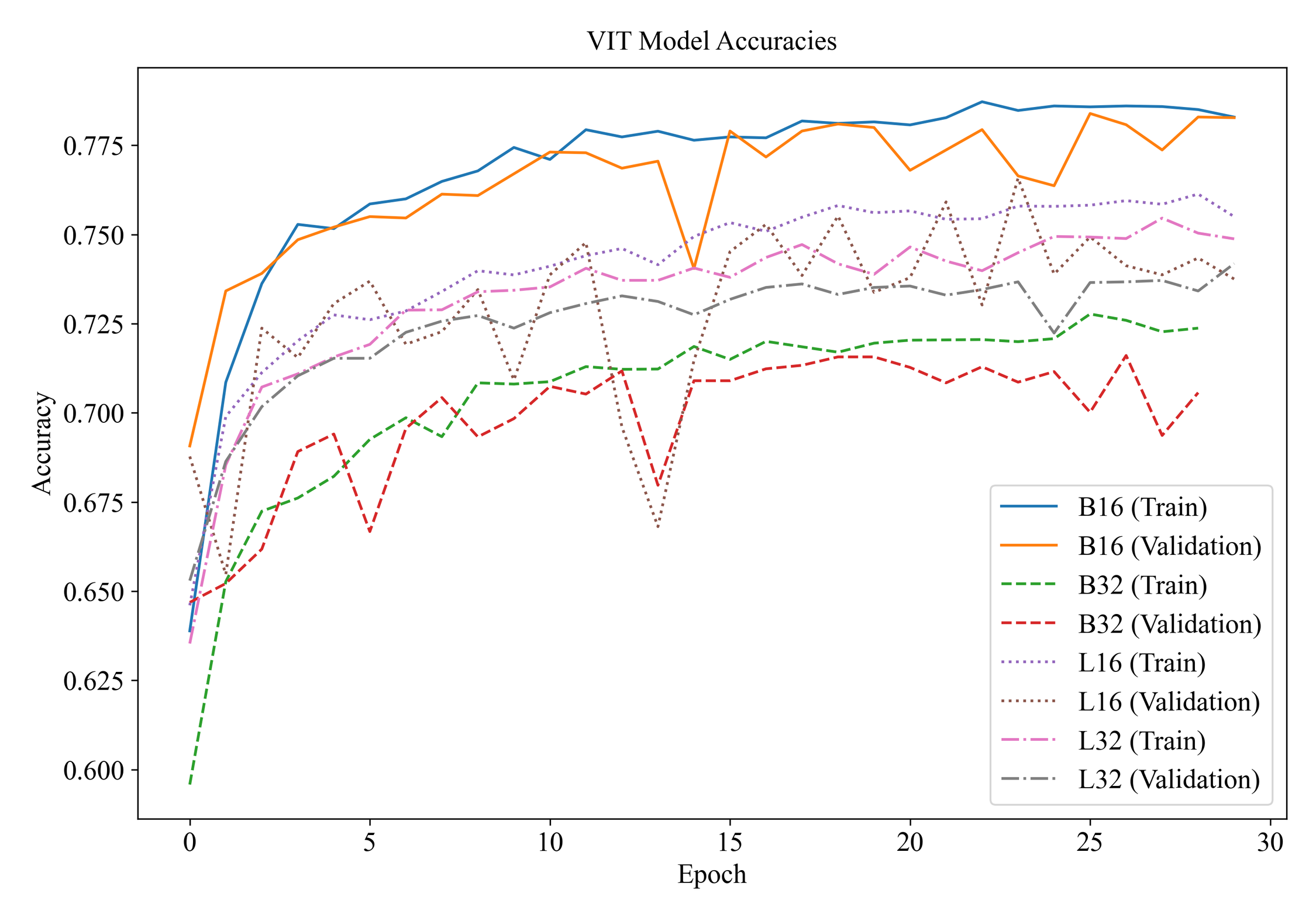

Supplement: S5 Fig — (TIF) [file pone.0288962.s005.tif]

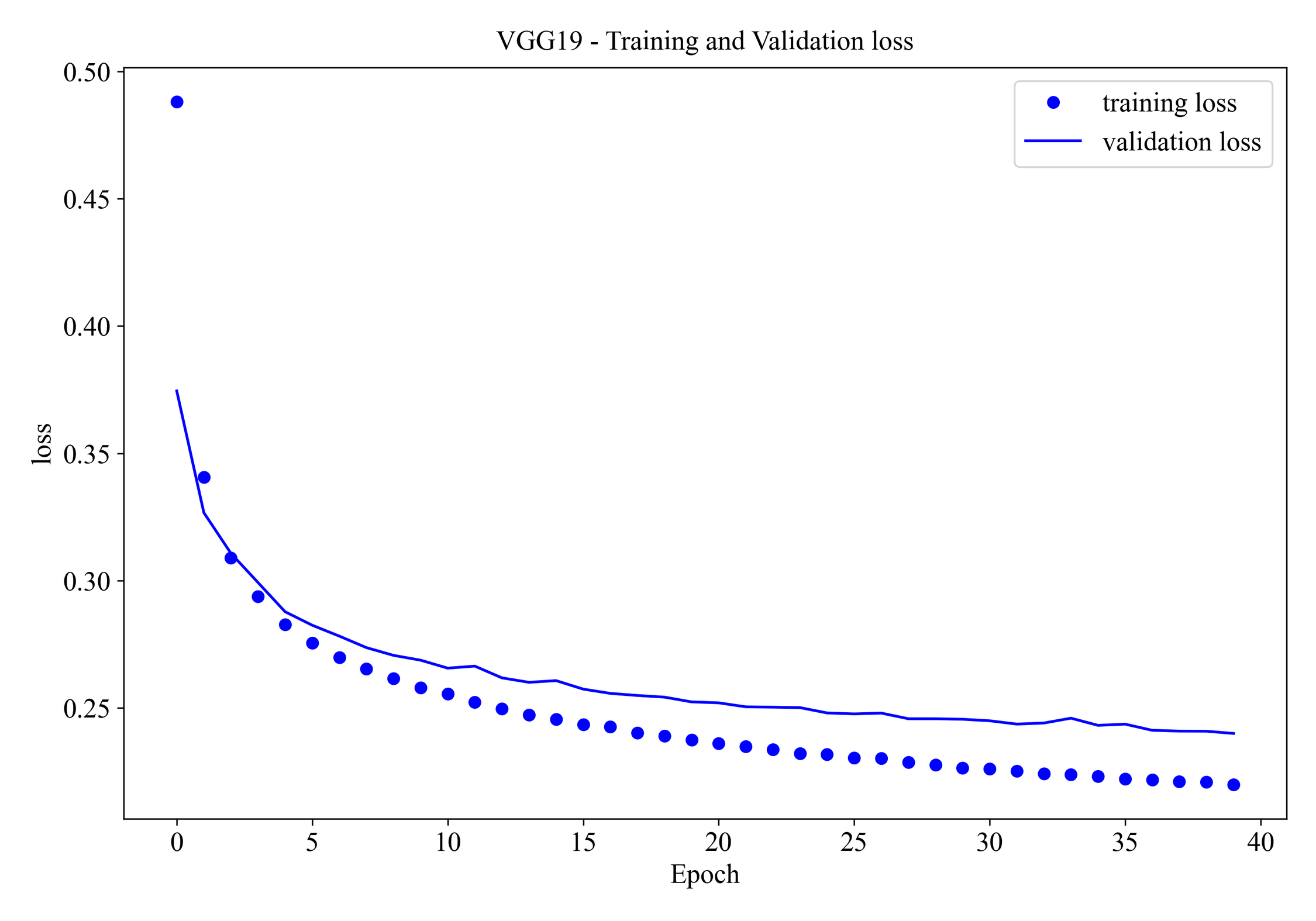

Supplement: S6 Fig — (TIF) [file pone.0288962.s006.tif]

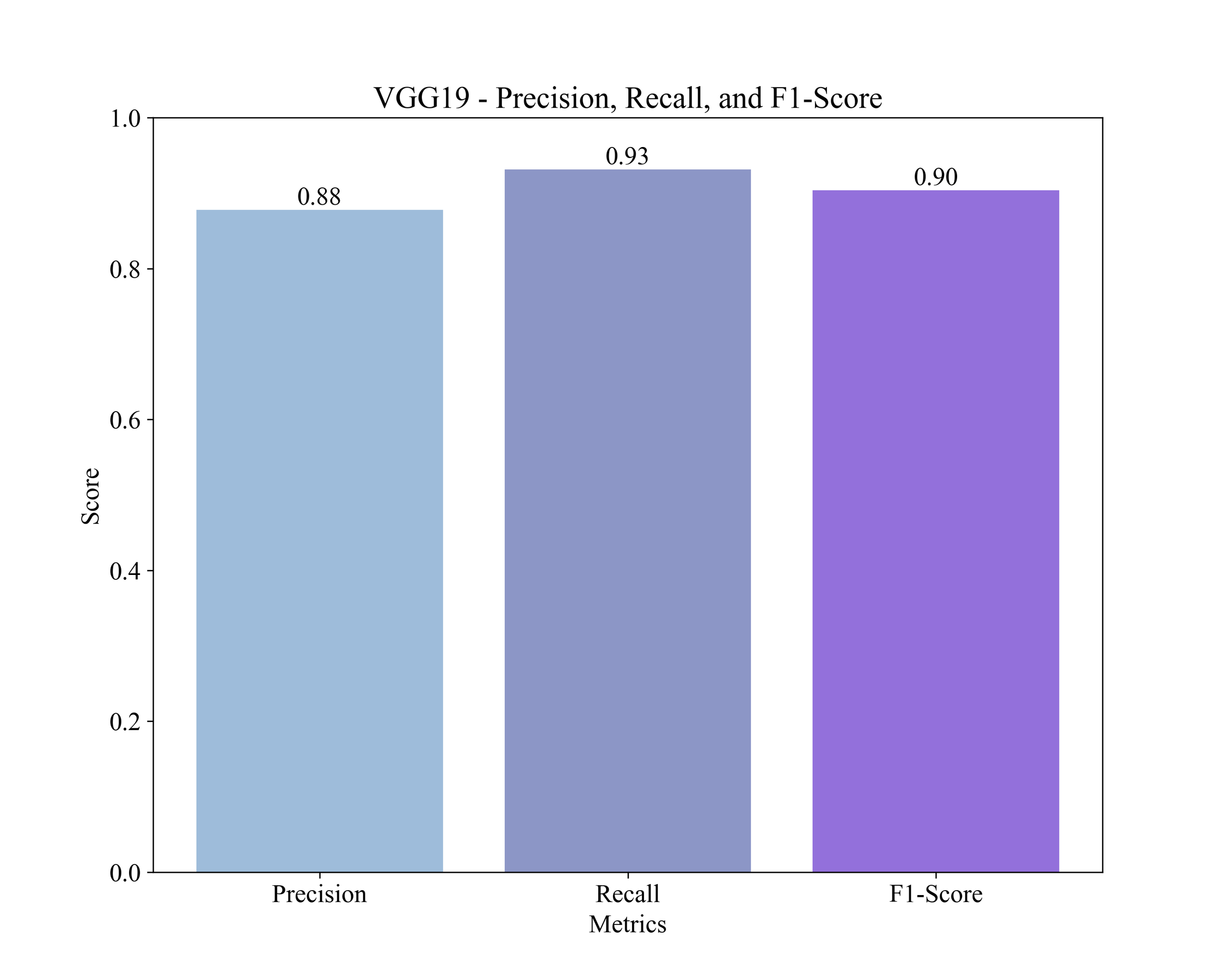

Supplement: S7 Fig — (TIF) [file pone.0288962.s007.tif]

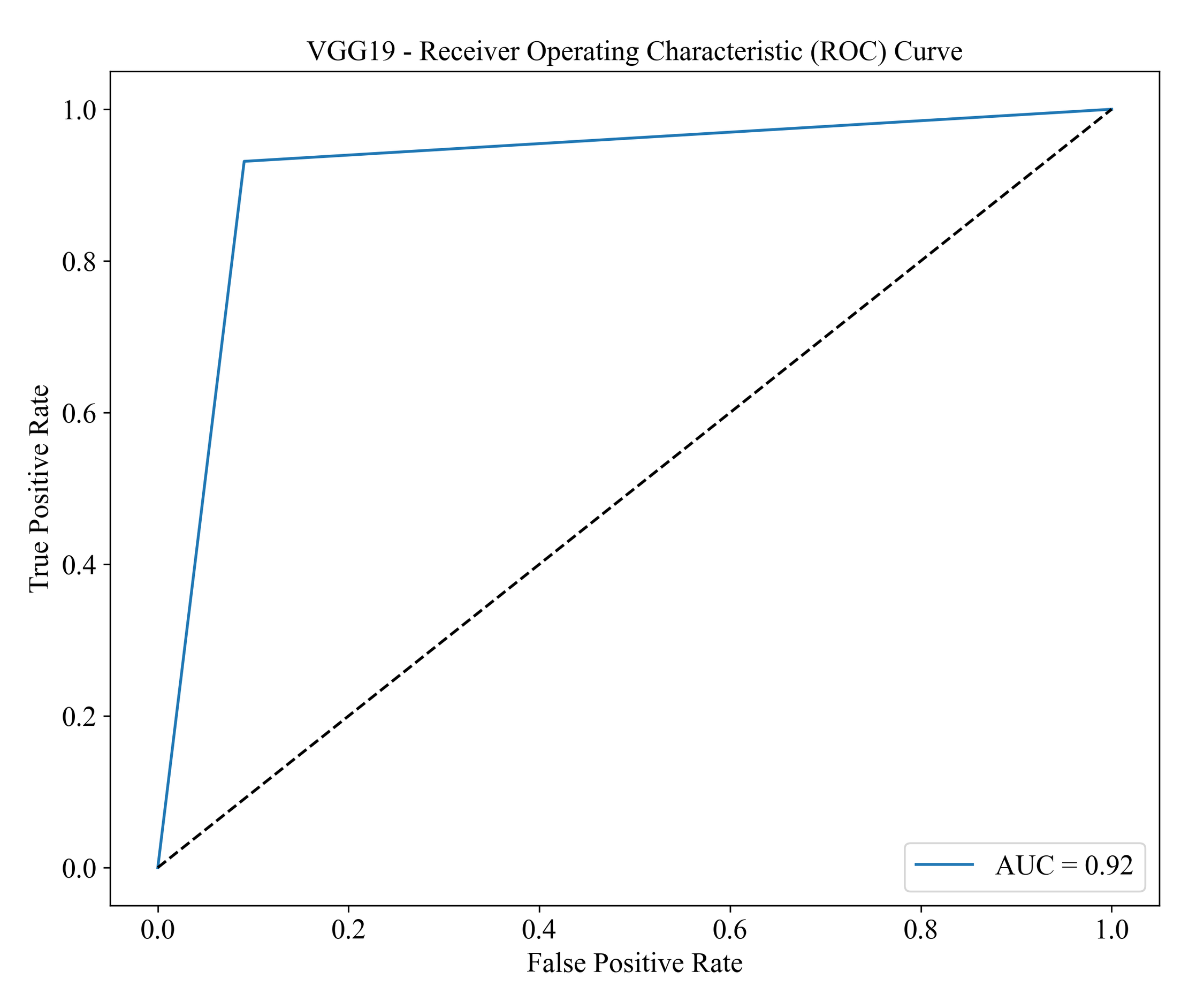

Supplement: S8 Fig — (TIF) [file pone.0288962.s008.tif]

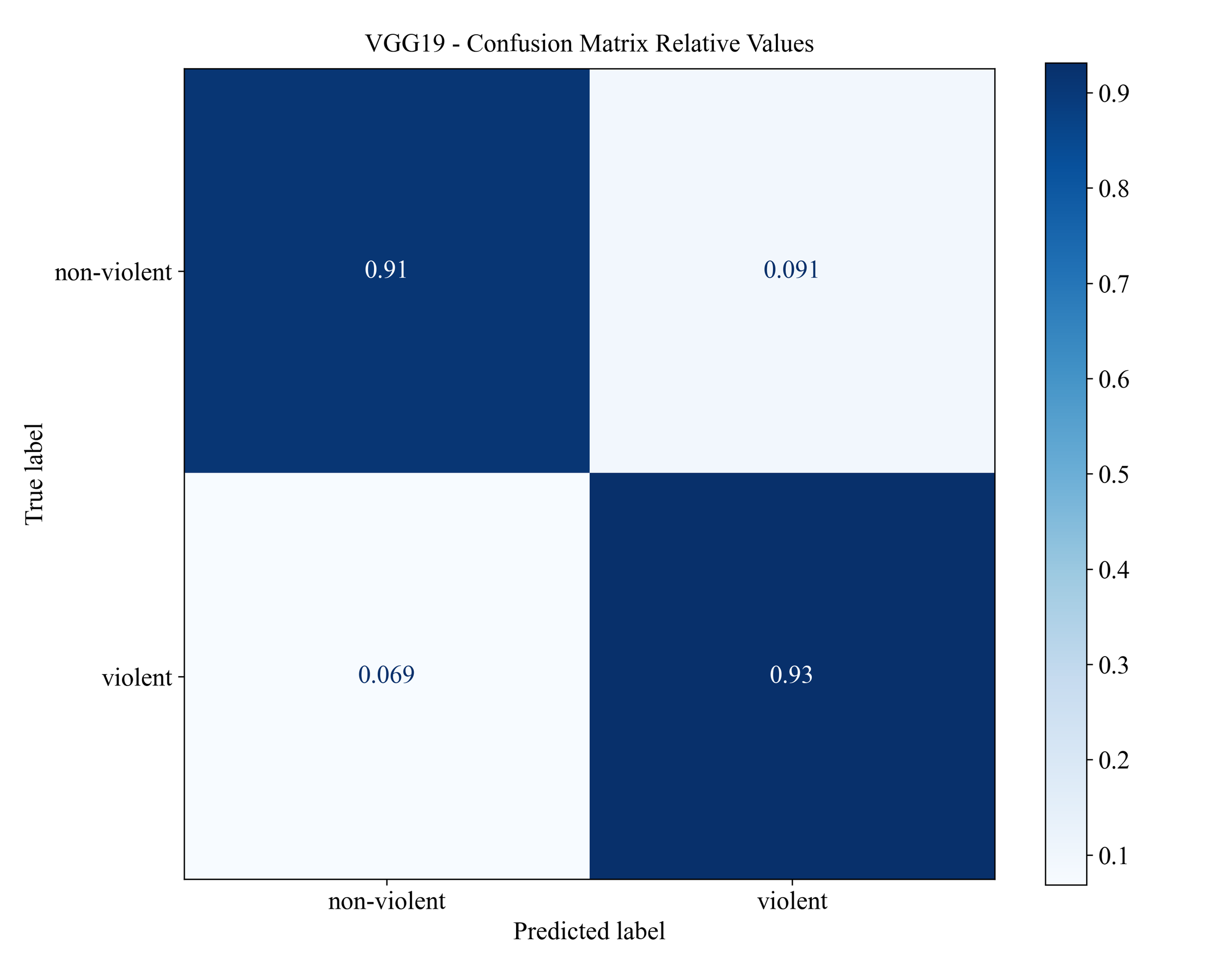

Supplement: S9 Fig — (TIF) [file pone.0288962.s009.tif]

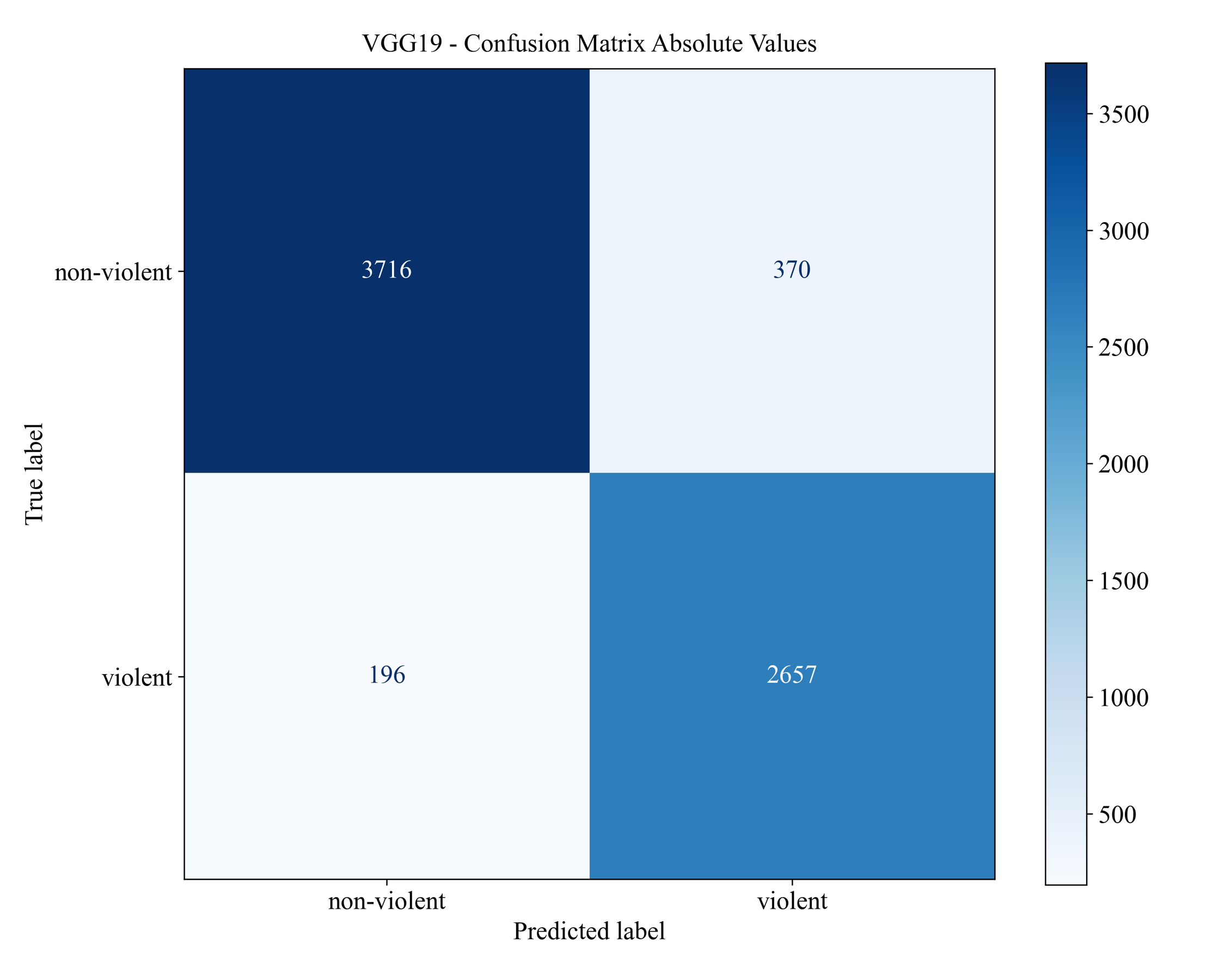

Supplement: S10 Fig — (TIF) [file pone.0288962.s010.tif]
