# Supplementary material for: Attention-grabbing news coverage: Violent images of the Black Lives Matter movement and how they attract user attention on Reddit
Source: PLoS One. 2023 Aug 9;18(8):e0288962. doi: 10.1371/journal.pone.0288962 (PMC10411814; doi:10.1371/journal.pone.0288962)
Supplement: S1 Table — (DOCX) [file pone.0288962.s011.docx]

**S1 Table. Descriptive overview of variables.**

| **Name** | **Kind** | **Type** | **Categories** | **Distribution** | **Retrieved** |
| --- | --- | --- | --- | --- | --- |
| total number of comments | dependent | interval | numeric | min = 1  max = 9,985  median = 8 | Reddit |
| violent image | independent | binary | 0 = nonviolent  1 = violent | nonviolent = 5,486  violent = 387 | VGG19 |
| BERT sentiment | control | binary | 0 = positive  1 = negative | positive = 1,253  negative = 4,620 | sentiment analysis with BERT model |
| political leaning | control | categorical | 0 = neutral  1= conservative  2 = conspiracy  3 = liberal | neutral = 793  conservative = 1,000  conspiracy = 2  liberal= 4,078 | mediabiasfactcheck.com |
| factual reporting | control | categorical | 0 = mixed  1 = high  2 = low | mixed = 2,886  high = 2,830  low = 157 | mediabiasfactcheck.com |
| traffic | control | categorical | 0 = medium  1 = high  2 = minimal | medium = 584  high = 5,244  minimal = 45 | mediabiasfactcheck.com |
| subreddit | control | categorical | 0 = politics  1 = news  2 = worldnews | politics = 4,324  news = 1,114  worldnews = 435 | Reddit |
| NSFW | control | binary | 0 = no NSFW tag  1 = NSFW tag | no NSFW tag = 5,870  NSFW tag = 3 | Reddit |
| link flair | control | binary | 0 = no link flair  1 = link flair | no link flair = 2,637  link flair = 3,236 | Reddit |
| number of cross-posts | control | interval | numeric | min = 0  max = 23 | Reddit |
| weekday/ weekend (US Pacific time) | control | binary | 0 = weekday  1 = weekend | weekday = 4,505  weekend = 1,368 | Reddit |
| time of day (US Pacific time) | control | categorical | 0 = afternoon/evening  1 = morning  2 = night | afternoon/evening = 2,395  morning = 2,417  night = 1,061 | Reddit |
| type of news outlet | control | categorical | 0 = newspaper  1 = magazine  2 = news agency  3 = organization/foundation  4 = radio station  5 = TV station  6 = website | newspaper = 1,986  magazine = 308  news agency = 240  organization/foundation = 63  radio station = 85  TV station = 1,953  website = 1,238 | mediabiasfactcheck.com |
