# Supplementary material for: Attention-grabbing news coverage: Violent images of the Black Lives Matter movement and how they attract user attention on Reddit
Source: PLoS One. 2023 Aug 9;18(8):e0288962. doi: 10.1371/journal.pone.0288962 (PMC10411814; doi:10.1371/journal.pone.0288962)
Supplement: S2 Table — (DOCX) [file pone.0288962.s012.docx]

**S2 Table. Own convolutional neural network architecture.**

| Model: Sequential | | |
| --- | --- | --- |
| Layer | Output Shape | Parameter |
| Convolutional | (None, 253, 253, 32) | 896 |
| Max Pooling | (None, 126, 126, 32) | 0 |
| Convolutional_1 | (None, 124, 124, 64) | 18,496 |
| Max Pooling_1 | (None, 62, 62, 64) | 0 |
| Convolutional_2 | (None, 60, 60, 128) | 73,856 |
| Dropout | (None, 60, 60, 128) | 0 |
| Max Pooling_2 | (None, 30, 30, 128) | 0 |
| Convolutional_3 | (None, 28, 28, 128) | 147,584 |
| Max Pooling_3 | (None, 14, 14, 128) | 0 |
| Flatten | (None, 25,088) | 0 |
| Dropout_1 | (None, 25,088) | 0 |
| Dense | (None, 512) | 12,845,568 |
| Dense_1 | (None, 1) | 513 |
| Total parameter: 13,086,913  Trainable parameters: 13,086,913  Non-trainable parameters: 0 | | |
