# Supplementary material for: Attention-grabbing news coverage: Violent images of the Black Lives Matter movement and how they attract user attention on Reddit
Source: PLoS One. 2023 Aug 9;18(8):e0288962. doi: 10.1371/journal.pone.0288962 (PMC10411814; doi:10.1371/journal.pone.0288962)
Supplement: S3 Table — (DOCX) [file pone.0288962.s013.docx]

**S3 Table. Negative binomial regression results of models with different datasets and variables (standard error in parentheses, p values in square brackets).**

|  | **model 1: complete data including zero comments** | **model 2: data without zero comments & with BLM in title variable** | **model 3: data without zero scores & total votes as dependent variable** |
| --- | --- | --- | --- |
|  | dependent count variable: number of comments | dependent count variable: number of comments | dependent count variable: total votes |
| VGG19 violent image | -0.096 (0.086) [0.264] | 0.014 (0.072) [0.845] | -0.006 (0.079) [0.936] |
| BERT sentiment negative | 0.104• (0.065) [0.035] |  | -0.283*** (0.052) [0.000] |
| BLM in title negative |  | 0.302*** (0.040) [0.000] |  |
| BLM in title positive |  | 0.212*** (0.059) [0.000] |  |
| political leaning conservative | -0.628*** (0.091) [0.000] | -0.203** (0.073) [0.006] | -0.911*** (0.089) [0.000] |
| political leaning conspiracy | -1.213 (0.776) [0.118] | -0.080 (0.990) [0.935] | -1.509* (0.724) [0.037] |
| political leaning liberal | -0.254*** (0.072) [0.001] | -0.048 (0.058) [0.406] | -0.130• (0.067) [0.051] |
| factual reporting high | 0.233*** (0.052) [0.000] | 0.091* (0.041) [0.029] | 0.565*** (0.048) [0.000] |
| factual reporting low | -0.709*** (0.136) [0.000] | -1.009*** (0.124) [0.000] | -1.246*** (0.131) [0.000] |
| traffic high | -0.541*** (0.083) [0.000] | -0.242*** (0.066) [0.000] | -0.639*** (0.079) [0.000] |
| traffic minimal | -0.200 (0.269) [0.459] | -1.221*** (0.217) [0.000] | -0.665** (0.257) [0.010] |
| subreddit news | -0.756*** (0.051) [0.000] | 0.996*** (0.048) [0.000] | -1.064*** (0.048) [0.000] |
| subreddit worldnews | -0.299*** (0.076) [0.000] | 0.619*** (0.071) [0.000] | -0.852*** (0.070) [0.000] |
| NSFW | -1.399* (0.586) [0.017] | -1.174 (0.798) [0.141] | -0.447 (0.525) [0.395] |
| link flair | -0.317*** (0.049) [0.000] | -0.569*** (0.037) [0.000] | -0.814*** (0.046) [0.000] |
| number of cross-posts | 1.377*** (0.022) [0.000] | 0.901*** (0.014) [0.000] | 1.732*** (0.019) [0.000] |
| weekend US pacific | 0.394*** (0.054) [0.000] | 0.237*** (0.042) [0.000] | 0.534*** (0.051) [0.000] |
| morning US pacific | -0.004 (0.049) [0.940] | -0.016 (0.039) [0.691] | -0.021 (0.046) [0.643] |
| night US pacific | 0.070 (0.063) [0.262] | -0.085• (0.050) [0.090] | 0.081 (0.058) [0.167] |
| type of news outlet magazine | 0.142 (0.115) [0.215] | 0.251** (0.086) [0.003] | 0.380*** (0.108) [0.000] |
| type of news outlet news agency | -0.252• (0.133) [0.058] | -0.741*** (0.102) [0.000] | -0.495*** (0.124) [0.000] |
| type of news outlet organization/foundation | -0.736** (0.236) [0.002] | -0.186 (0.182) [0.307] | -0.862*** (0.227) [0.000] |
| type of news outlet radio | -0.350• (0.197) [0.076] | -0.338* (0.154) [0.028] | -0.761*** (0.179) [0.000] |
| type of news outlet TV station | 0.475*** (0.057) [0.000] | 0.044 (0.047) [0.341] | 0.139* (0.054) [0.010] |
| type of news outlet website | -0.258*** (0.064) [0.000] | 0.061 (0.052) [0.236] | -0.263*** (0.060) [0.000] |
| Constant | 3.358*** (0.121) [0.000] | 3.218*** (0.089) [0.000] | 6.067*** (0.113) [0.000] |
| Observations | 9,729 | 5,873 | 8,917 |
| Null deviance | 15,007.1 on 9,728 df | 14,208.2 on 5,872 df | 21,675 on 8,916 df |
| Res. deviance | 9,859.7 on 9,705 df | 6,894.7 on 5,848 df | 11,726 on 8,893 df |
| AIC | 60,451 | 50,386 | 86,704 |
| Theta | 0.213 | 0.561 | 0.263 |
| Std. Error | 0.003 | 0.009 | 0.003 |
| Note: •p<0.1; *p<0.05; **p<0.01, ***p<0.001 | | | |
