# Supplementary material for: Attention-grabbing news coverage: Violent images of the Black Lives Matter movement and how they attract user attention on Reddit
Source: PLoS One. 2023 Aug 9;18(8):e0288962. doi: 10.1371/journal.pone.0288962 (PMC10411814; doi:10.1371/journal.pone.0288962)
Supplement: S1 File — (DOCX) [file pone.0288962.s014.docx]

S1 File. Appendix – Attention-grabbing news coverage: Violent images of the Black Lives Matter movement and how they attract user attention on Reddit

Theresa Henn^1^, Oliver Posegga^1*^

^1^Department of Information Systems and Social Networks, University of Bamberg, Bamberg, Germany

^*^Corresponding author: E-mail: oliver.posegga@uni-bamberg.de

Table of Contents

[Data availability statement 3](#_Toc140061026)

[Descriptive overview of variables 8](#_Toc140061027)

[Sentiment model 10](#_Toc140061028)

[Image classifiers 13](#_Toc140061029)

[Negative binomial regression models 22](#_Toc140061030)

[References 31](#_Toc140061031)

# **Data availability statement**

In the following, we will describe in detail the different datasets utilized within our paper to make our analysis transparent and replicable.

The paper is based on **four** different datasets:

- The first dataset is the **training dataset** for the image classifier and consists of 7,972 violent images and 8,986 nonviolent images. We obtained these images partially from Won et al.’s [1] “UCLA Protest Image Dataset,” a prelabeled protest-related dataset comprising more than 40,764 images. Besides this data source, we enriched our training dataset with pictures from a Black Lives Matter (BLM) related keyword search on Bing. This dataset was then used to train our image classifier to classify images as violent (1) or nonviolent (0).
- The second dataset is the **test dataset** and was likewise obtained through a combination of images of the dataset provided by Won et al. [1] and images from a keyword search related to the Black Lives Matter movement. Combining both data sources, this dataset contains 2,854 violent and 4,088 nonviolent pictures. It was used to evaluate the performance of the image classifier.
- The third dataset, the **NewsMTSC dataset**, was introduced by Hamborg and Donnay [2] as a labeled dataset for sentiment classification in political news articles. We used their dataset’s real-world (rw) sentiment distribution subset, which contains 8,740 train samples, 343 validation samples, and 803 test samples of news sentences experts annotated as positive, negative, or neutral. We used this dataset to train our BERT model to classify submission titles as positive or negative.
- The fourth dataset is the **Reddit BLM dataset** obtained from Reddit via the Pushshift submission dumps. After filtering the data and downloading images attached to the submissions’ URLs, we received a final dataset of 5,873 observations. Based on this dataset, we conducted the negative binomial regression analysis presented in the manuscript.

To receive these final datasets, we proceeded as follows:

- To reduce Won et al.’s [1] protest dataset to a training dataset appropriate for our analysis, we first transformed their continuous violence codes (ranging from 0 “nonviolent” to 1 “very violent”) into a binary code (0 “nonviolent” and 1 “violent”). We classified every original protest picture score above 0.5 as “violent.” Using all images classified as violent, we created a balanced sample by adding the same number of nonviolent pictures (with the smallest original violence scores, all close to 0). Thus, our training dataset contained 1,283 violent and 1,283 nonviolent images. We manually reviewed all images and adjusted the violence score if necessary to guarantee that this binary classification conforms with our definition of violence (adopted from Rucht [3]). We extended this sample and included additional images identified via the search engine Bing based on the following 25 keywords in English and German: Black Lives Matter; BLM violen*; police brutality; protest violence; police violence; Protest Gewalt; Black Lives Matter demonstration; Polizei Gewalt; George Floyd; Rayshard Brooks; Donald Trump; Joe Biden; burning; protest shooting; violent demonstration; Hong Kong violence; Portland protest; politicians; mug shot; American politicians; property destruction protest; USA violent protest; protest hurt people; bleeding protest; dead protestors. Based on our definition of violence, we manually extracted 173 additional violent and 692 nonviolent images from the search results. Combining these images with our previous images obtained from the UCLA Protest Image Dataset, we ended up with 1,456 violent and 1,975 nonviolent images. We applied image augmentation to increase our sample size, resulting in 7,972 violent images and 8,986 nonviolent images as our final training dataset.
- The test dataset was created similarly. After selecting the most and least violent images from the test UCLA Protest Image Dataset, we received 350 violent and 350 nonviolent images. Again, we validated and adjusted the violence classification of the extracted images based on Rucht’s [3] violence definition and added pictures from the Bing search described above. At this stage, the dataset comprised 309 violent and 478 nonviolent images. After augmenting this data, the final test dataset contained 2,854 violent and 4,088 nonviolent images.
- Since we are interested in differentiating between positive and negative sentiment in news titles, we reduced the NewsMTSC dataset [2] to news sentences labeled as negative or positive. Doing so, we received 3,316 negative and 2,395 positive samples for our training data, 143 negative and 73 positive samples for the validation data, and 295 negative and 189 positive samples for our test data.
- For the Reddit BLM dataset, we downloaded the Pushshift submission dumps for May 2020 until May 2021 (105GB) on May 9, 2023, and filtered the data regarding the three subreddit of interest r/worldnews, r/politics, and r/news (830MB) which led to 1,887,202 submissions. After reducing the data towards the period of analysis from May 25, 2020, the day of George Floyd’s murder, until May 25, 2021, we received 1,763,659 submissions, of which r/news contained 878,812 submissions, r/politics 445,051 submissions, and r/worldnews 439,796 submissions. We removed all submissions whose titles did not match one of the three keywords, “black lives matter,” “george floyd,” and “blm,” resulting in a total of 17,083 submissions relevant to our analysis. Note that we also tested how an increased keyword set related to our analysis topic would increase our data corpus. We therefore added the keywords “police violence,” “breonna taylor,” and “rayshard brooks” to our keyword set. As we only received 3,008 additional submissions after doubling the keyword set while also introducing more noise to our data (e.g., police violence related to Covid-19 protests), we decided to continue working with our initial keyword set, as it showed to capture the relevant submissions for our analysis. For our data cleaning process, we removed a particular type of submission from our dataset, so-called “megathreads,” reducing the number of submissions to 17,069. For these submissions, we attempted to receive information regarding the categorical control variables “political leaning” (*H4*), “factual reporting” (*H5*), “traffic” (*H6*), and “type of news outlet” from the website mediabiasfactcheck.com. If we did not find all information on the news outlet, we coded the observation as missing and dropped the submission. Out of the remaining 11,813 submissions, we obtained 9,373 images attached to the linked news outlets through the Python library “Newspaper3k.” Thus, we dropped 2,440 submissions for which we could not get an image or from which the image was broken. As we were interested in submissions that at least received a minimum of attention, we deleted all submissions that received zero comments ending up with 5,876 observations. After dropping all missing values of the other variables related to our analysis, we received 5,873 submissions. Thus, our final Reddit BLM dataset comprised 5,873 submissions, including one image per submission. These 5,873 submissions were posted by 2,091 unique users (note that 1,975 submissions were posted by authors that were deleted by the time of data retrieval) and included 4,833 unique URLs; 4,324 submissions were posted in r/politics, 1,114 in r/news, and 435 in r/worldnews. Figure S1 shows a monthly distribution of submissions within our Reddit BLM dataset.

**S1 Figure. Histogram of monthly distribution of Reddit BLM submissions.**


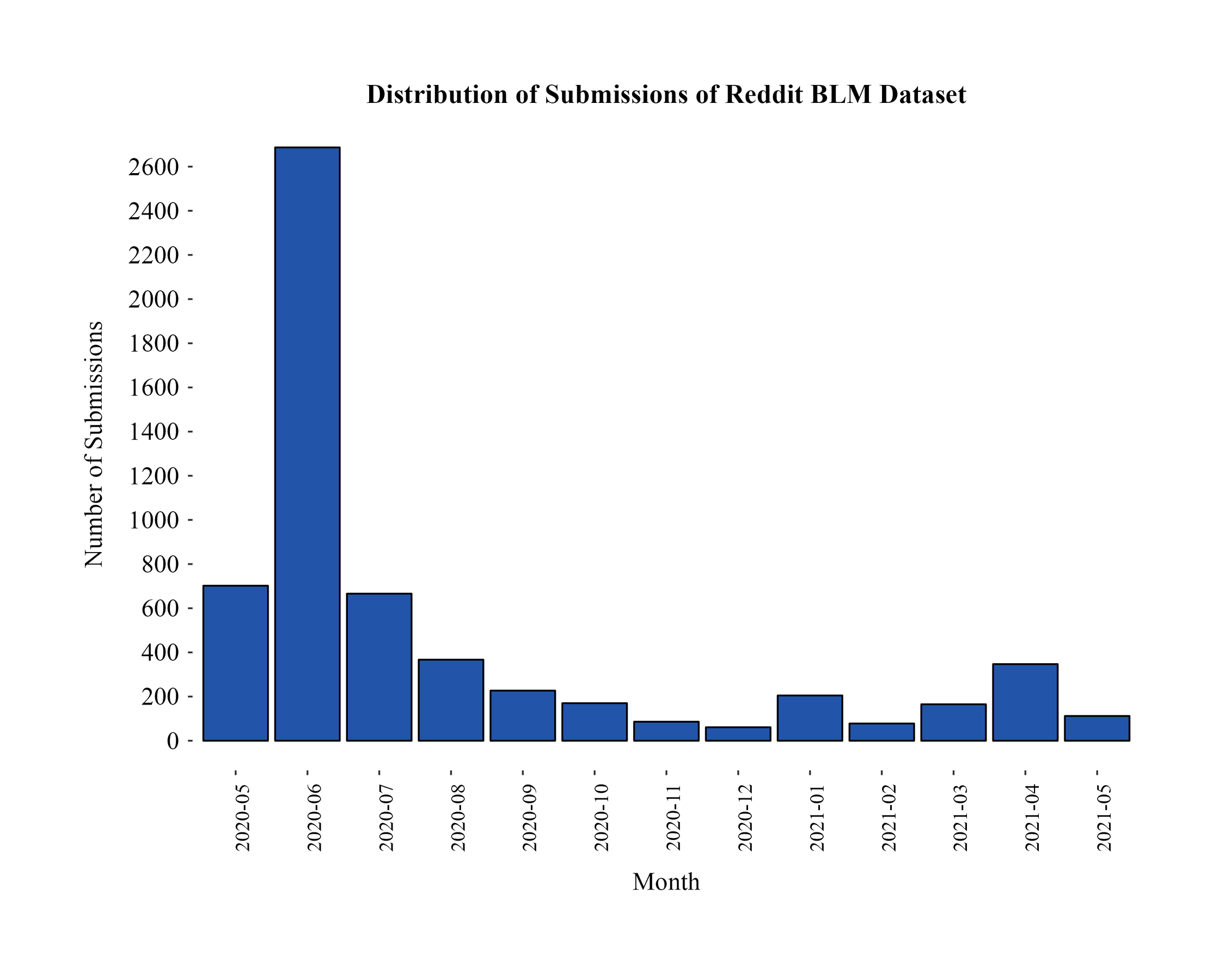


The primary Reddit BLM dataset underlying our model is available via OSF (<https://osf.io/4dr2x/?view_only=39ff5cb39c254b6c93eb12f9c205a941>). Since the data (violent and nonviolent images) used in our training and test dataset is considered third-party data belonging to Won et al. [1], we cannot share this data publicly. Yet, all information to obtain and prepare the dataset can be obtained from their public GitHub repository [4]. By applying the different data transformation steps mentioned above, our datasets can be reconstructed, and results can be replicated. The NewsMTSC dataset from Hamborg and Donnay [2] is available via Hugging Face [5].

# **Descriptive overview of variables**

This section provides a descriptive overview of the variables used within our negative binomial regression analysis (see S1 Table). Additionally, we show the distribution of our dependent variable number of comments, which is highly positively skewed (see S2 Figure).

**S1 Table. Descriptive overview of variables.**

| **Name** | **Kind** | **Type** | **Categories** | **Distribution** | **Retrieved** |
| --- | --- | --- | --- | --- | --- |
| total number of comments | dependent | interval | numeric | min = 1  max = 9,985  median = 8 | Reddit |
| violent image | independent | binary | 0 = nonviolent  1 = violent | nonviolent = 5,486  violent = 387 | VGG19 |
| BERT sentiment | control | binary | 0 = positive  1 = negative | positive = 1,253  negative = 4,620 | sentiment analysis with BERT model |
| political leaning | control | categorical | 0 = neutral  1= conservative  2 = conspiracy  3 = liberal | neutral = 793  conservative = 1,000  conspiracy = 2  liberal= 4,078 | mediabiasfactcheck.com |
| factual reporting | control | categorical | 0 = mixed  1 = high  2 = low | mixed = 2,886  high = 2,830  low = 157 | mediabiasfactcheck.com |
| traffic | control | categorical | 0 = medium  1 = high  2 = minimal | medium = 584  high = 5,244  minimal = 45 | mediabiasfactcheck.com |
| subreddit | control | categorical | 0 = politics  1 = news  2 = worldnews | politics = 4,324  news = 1,114  worldnews = 435 | Reddit |
| NSFW | control | binary | 0 = no NSFW tag  1 = NSFW tag | no NSFW tag = 5,870  NSFW tag = 3 | Reddit |
| link flair | control | binary | 0 = no link flair  1 = link flair | no link flair = 2,637  link flair = 3,236 | Reddit |
| number of cross-posts | control | interval | numeric | min = 0  max = 23 | Reddit |
| weekday/ weekend (US Pacific time) | control | binary | 0 = weekday  1 = weekend | weekday = 4,505  weekend = 1,368 | Reddit |
| time of day (US Pacific time) | control | categorical | 0 = afternoon/evening  1 = morning  2 = night | afternoon/evening = 2,395  morning = 2,417  night = 1,061 | Reddit |
| type of news outlet | control | categorical | 0 = newspaper  1 = magazine  2 = news agency  3 = organization/foundation  4 = radio station  5 = TV station  6 = website | newspaper = 1,986  magazine = 308  news agency = 240  organization/foundation = 63  radio station = 85  TV station = 1,953  website = 1,238 | mediabiasfactcheck.com |

**S2 Figure. Histogram of dependent variable number of comments.**


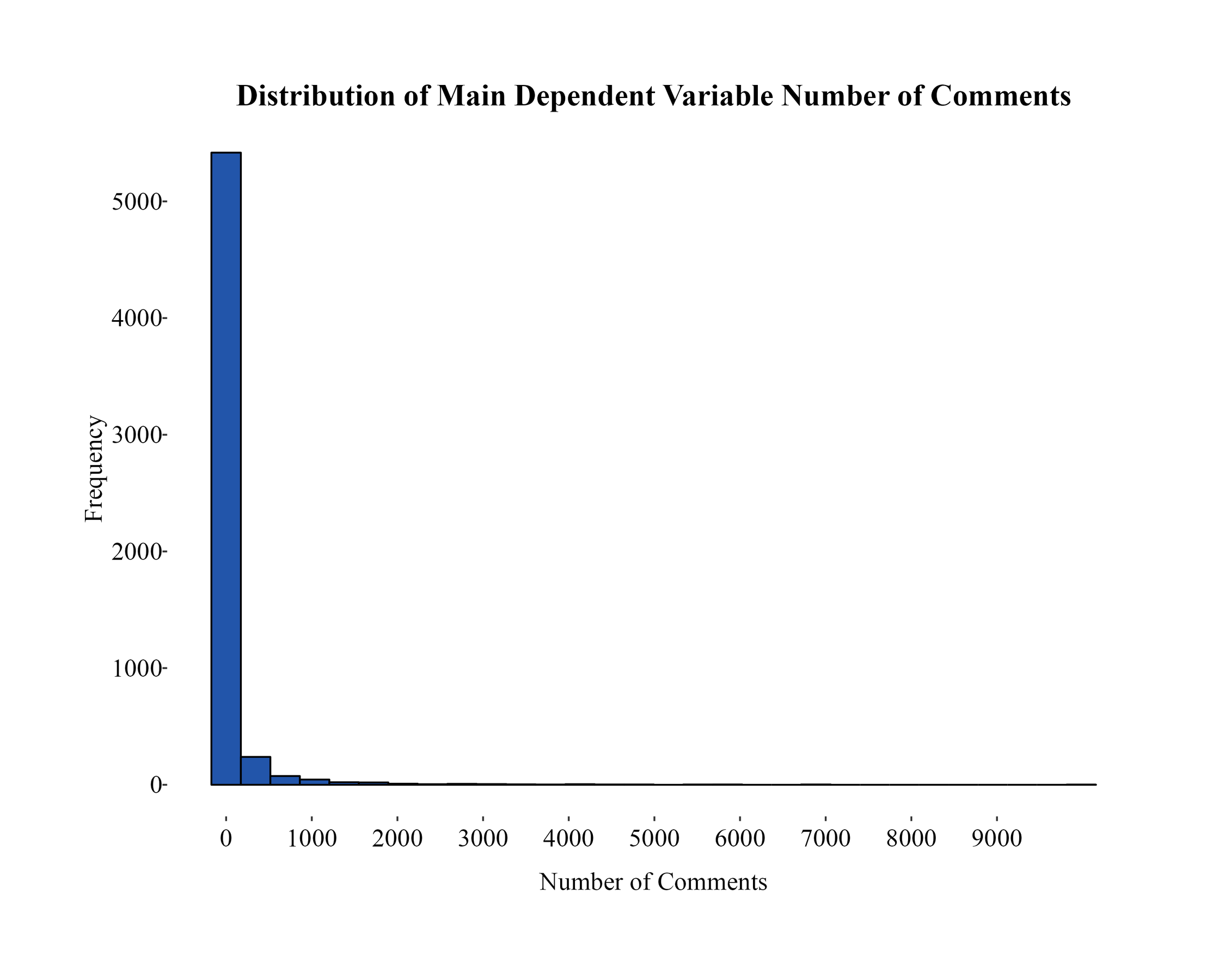


# **Sentiment model**

For our sentiment analysis of submission titles, we used the “BERT base model uncased,” derived from the Hugging Face platform [6]. The model is trained on English Wikipedia and the BookCorpus data [7] and contains 110 million parameters. To customize the model for classifying the sentiment of news articles, we trained the model with the NewsMTSC dataset [2], a labeled dataset for sentiment classification in political news articles containing the categories positive, negative, and neutral. Since we were interested in positive and negative sentiment classification, we dropped the neutral samples from the training, validation, and test dataset. We used the remaining 5,711 samples as training data and the remaining 207 samples as validation data. We compiled the model with the adam optimizer, a sparse categorical cross-entropy loss function, and a learning rate of 0.00002. We implemented an early stop callback function with a patience parameter of three, monitoring the validation accuracy. Thus, the model stops the training if the validation accuracy does not improve for three consecutive epochs. We started the training process with ten epochs, but due to the early stop callback function, the training process stopped after three epochs. The model received a training accuracy of 99.17% and a validation accuracy of 87.66% (see S3 Figure). When applying the model to the test dataset, it received an accuracy of 87.40%.

**S3 Figure. Training and validation accuracy of BERT model.**


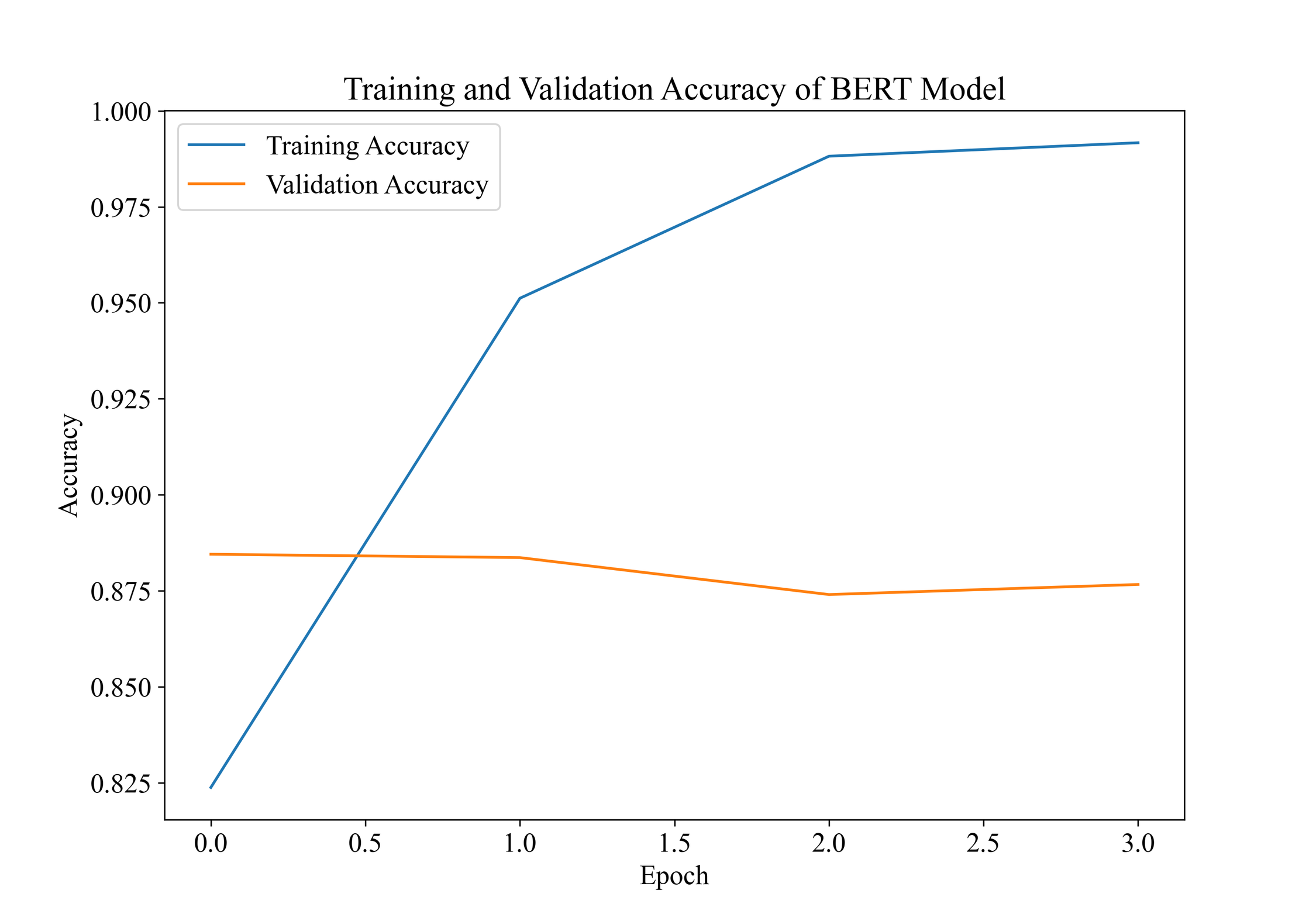


To evaluate the performance of the trained BERT model on our Reddit BLM dataset, we drew a random sample of 200 submission titles. We manually labeled them as either positive or negative. The manual and automatic classification matched 168 out of 200 cases (84.0%), which aligns with the previous results on the testing and validation data.

To contrast this result with another sentiment analysis approach, we also tested the performance of the Python library “Valence Aware Dictionary and Sentiment Reasoner” (VADER) on our data. We chose VADER as a second sentiment analysis approach because it has been shown to perform exceptionally well on news data and Reddit [8]. VADER bases its annotations on prelabeled dictionaries with sentiment scores for each listed record. By relating the polarity scores (the amount of negative and positive sentiment within the text) to the computed intensity scores (the strength of the respective emotion), VADER calculates negative, positive, and neutral values for each tested observation. It then blends these values into a single compound score ranging from -1 (very negative) to 1 (very positive). We used this compound value as a threshold by dichotomously labeling a title with a score <0 as “negative” and each title with a score >=0 as “positive.” When comparing the sentiment prediction of VADER on a random sample of 200 submission titles of our Reddit BLM dataset with our manual labeling, 154 out of 200 samples (77.0%) were correctly classified.

Since the BERT model outperformed the VADER classification while also considering the context of a sentence compared to a bag-of-words approach, we utilized our trained BERT model for the analysis in the manuscript.

# **Image classifiers**

For our image analysis, we trained and validated different state-of-the-art convolutional neural network (CNN) and vision transformer (VIT) models with our training dataset to detect the best-fitting architecture for our classification context.

For the CNN models, we used four different architectures: (1) a self-created CNN architecture (see S2 Table) which we built with the Python deep learning libraries “TensorFlow” and “Keras” and trained from scratch, (2) a VGG19 architecture, (3) a ResNet50 architecture, and (4) an EfficientNetB4 architecture which were derived from TensorFlow and which were pre-trained on the ImageNet-1k dataset which comprises 1,000 different image classes and more than 1.2 million images [9]. We decided on these models as the latter three represent state-of-the-art architectures for image classification with CNNs [see, e.g., 10,11]. We decided on the former as it achieved convincing classification results (see S4 Figure) while utilizing fewer parameters than the other models, resulting in a faster training process (see S2 Table for self-created CNN’s architecture and the number of parameters).

**S2 Table. Own convolutional neural network architecture.**

| Model: Sequential | | |
| --- | --- | --- |
| Layer | Output Shape | Parameter |
| Convolutional | (None, 253, 253, 32) | 896 |
| Max Pooling | (None, 126, 126, 32) | 0 |
| Convolutional_1 | (None, 124, 124, 64) | 18,496 |
| Max Pooling_1 | (None, 62, 62, 64) | 0 |
| Convolutional_2 | (None, 60, 60, 128) | 73,856 |
| Dropout | (None, 60, 60, 128) | 0 |
| Max Pooling_2 | (None, 30, 30, 128) | 0 |
| Convolutional_3 | (None, 28, 28, 128) | 147,584 |
| Max Pooling_3 | (None, 14, 14, 128) | 0 |
| Flatten | (None, 25,088) | 0 |
| Dropout_1 | (None, 25,088) | 0 |
| Dense | (None, 512) | 12,845,568 |
| Dense_1 | (None, 1) | 513 |
| Total parameter: 13,086,913  Trainable parameters: 13,086,913  Non-trainable parameters: 0 | | |

For the self-created CNN architecture, we activated the last dense layer with a sigmoid function, which returns a number between 0 and 1 and is, therefore, suitable for our binary image classification. For compiling the self-created CNN, we deployed the optimizer root mean square prop (RMSprop) algorithm with a learning rate of 0.0001 and a binary cross-entropy (BCE) loss function. We started the model’s training by splitting the training dataset into 70% training images and 30% validation images. We also implemented an early stop callback function with a minimum delta of 0.1% and a patience parameter of 10 within our training process to prevent overfitting. Thus, the training stops if the validation loss does not improve within ten epochs by at least 0.1% compared to its best value. We initially implemented 40 training epochs with a batch size of 32, stopping at epoch 23 due to our early stop callback function (see S4 Figure).

For the VGG19 model, we froze all layers but the last fully connected layer of the pre-trained model, as unfreezing more layers did not yield better training results. We replaced the last fully connected layer with a dense layer that applies a sigmoid function as an activation. We compiled the model with the adam optimizer, a learning rate of 0.001, and a BCE loss function. We split the training data into 70% training images and 30% validation images. Likewise, we implemented an early stop callback function with a minimum delta of 0.1% and a patience parameter of 10. We started training the model with 40 epochs and a batch size of 32. As the model’s validation loss continuously improved by at least 0.1%, the training ended after 40 epochs (see S4 Figure).

For the ResNet50 and EfficientNetB4 architectures, we applied the same steps and parameters described for the VGG19 model. We solely unfroze the last 50 layers of the EfficientNetB4 model, as this approach yielded the best accuracy results when training this model (for an overview of the model accuracies, see S4 Figure).

**S4 Figure. Accuracy of CNN models.**


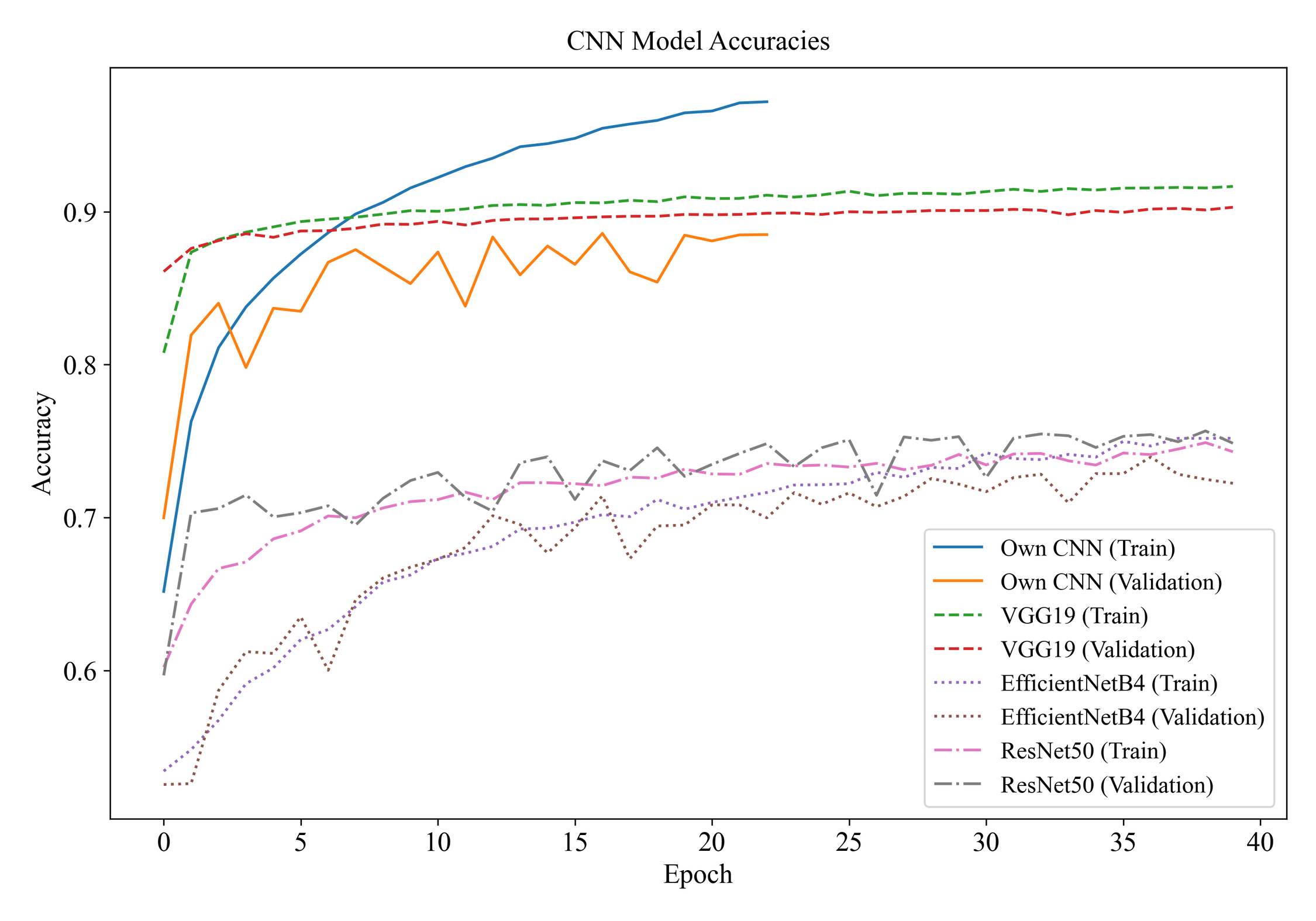


VIT models are considered one of the latest developments in computer vision by achieving equivalent or better results compared to state-of-the-art CNNs [see, e.g., 12,13]. Thus, we utilized the Keras implementation of four commonly applied and pre-trained VIT architectures and used our training data for the transfer learning [12]. The four architectures are (1) a VIT base model with an input patch size of 16x16 (B16), (2) a VIT base model with an input patch size of 32x32 (B32), (3) a VIT large model with an input patch size of 16x16 (L16), and (4) a VIT large model with an input patch size of 32x32 (L32). Note that these models’ configurations are directly adopted from the BERT model architecture [for more information, please see 12]. All four models are pre-trained on the ImageNet-21k dataset, which comprises more than 14 million images and nearly 22,000 different image classes [9]. We removed the last dense layer for all four models and replaced it with a dense layer activated by a sigmoid function. We also included an early stop callback function monitoring the validation loss with a patience parameter of 10 and a minimum delta of 0.1% to prevent overfitting. We compiled all four models with the adam optimizer and a BCE loss function. For training all four models, we split the training data into 70% training and 30% validation data and initiated the training process with 30 epochs and a batch size of 32. As the validation loss continuously improved by at least 0.1% during training for models B16, L16, and L32, the training ended at epoch 30; for model B32, the training ended at epoch 29 (see S5 Figure).

**S5 Figure. Accuracy of VIT models.**


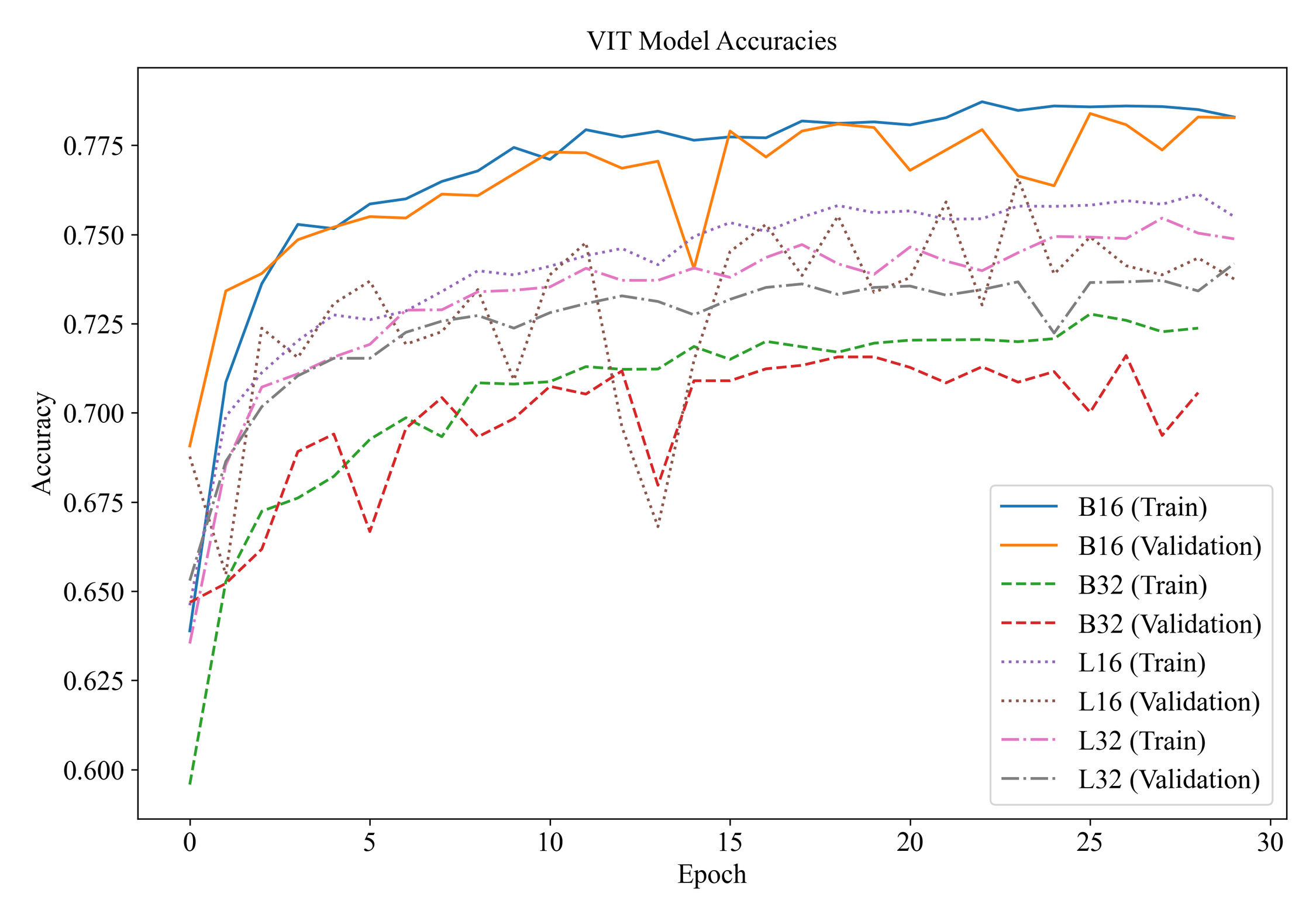


When comparing the accuracy levels of the training and validation data of the four CNNs and the four VIT models (see S4 Figure and S5 Figure), it becomes evident that our self-created CNN and the VGG19 architecture outperform the remaining CNNs, EfficientNetB4 and ResNet50 as well as all four VIT models. We thus continued working with those two models and tested how well they performed on our image data from the Reddit BLM dataset. We, therefore, classified 200 randomly selected images with both models. When manually evaluating the classification accuracy of the self-created CNN with our manual coding, out of 200 images, 171 (85.5%) were correctly classified as either violent or nonviolent. In comparison, when manually evaluating the performance of the VGG19 model on 200 randomly drawn samples from our Reddit BLM dataset, 180 out of 200 (90.0%) images were correctly classified. We, therefore, chose the VGG19 model as our image classifier for the classification of the Reddit BLM dataset.

Since the VGG19 model is our model of choice, we also evaluated the model’s performance regarding additional performance indicators. The model achieved an accuracy value of 91.65% on the training data and 90.29% on the validation data (see S4 Figure). When assessing the performance on the test dataset, the classifier achieved an accuracy of 91.84%. When evaluating the model’s training and validation loss during the training process (see S6 Figure), both curves continuously decreased, converging towards the x-axis. This finding indicates that the model picks up essential image features without overfitting the data.

**S6 Figure. VGG19 model loss function.**


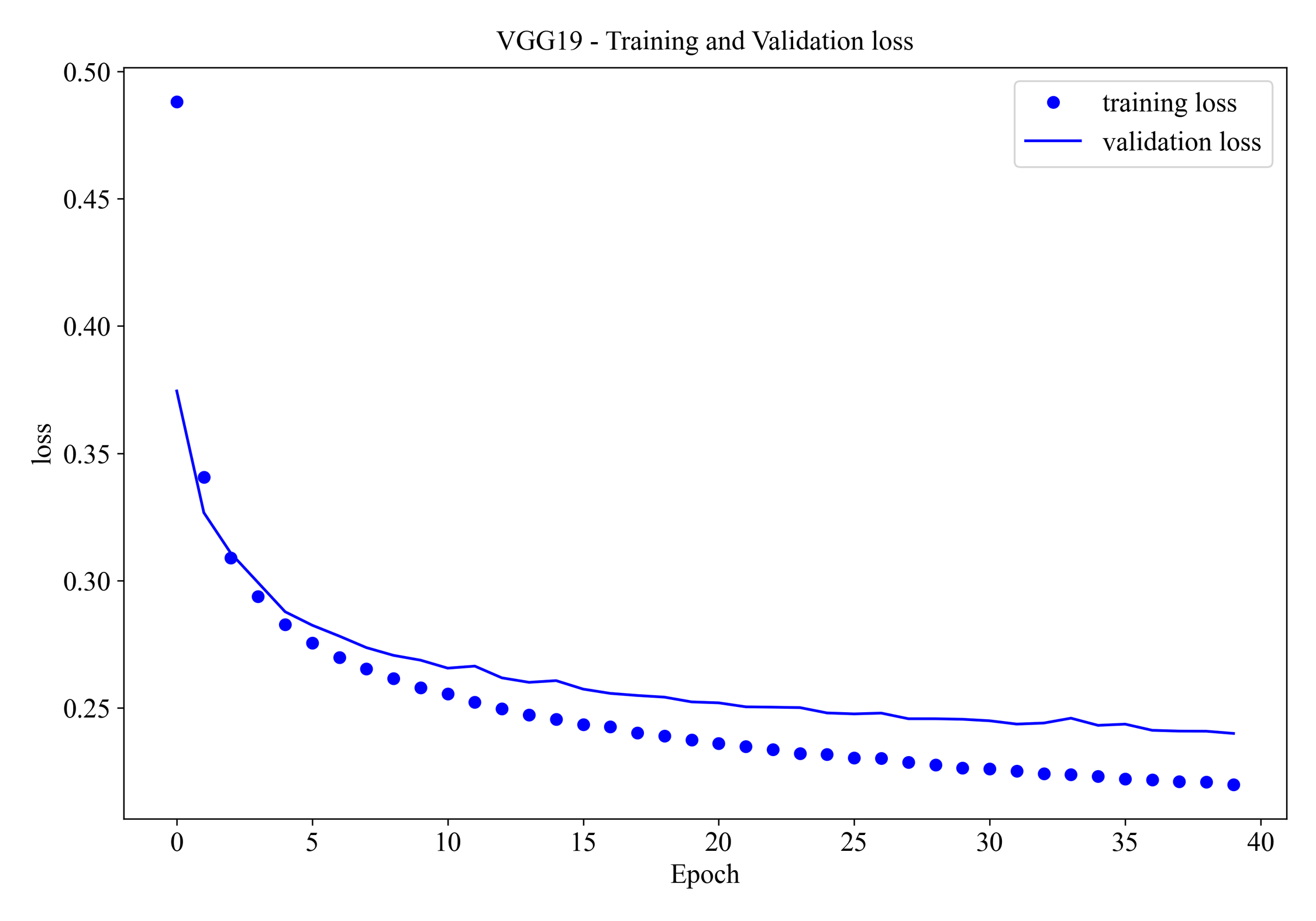


The VGG19 model received a precision score of 87.78%, a recall score of 93.13%, and an F1-score of 90.37% on the test dataset (see S7 Figure). Thus, the model can correctly classify violent images (precision) and detect most of the violent images within the dataset (recall). This outstanding performance on both levels is expressed by the very good F-1 score of 90.37%.

**S7 Figure. VGG19 model precision, recall, and F1-Score.**


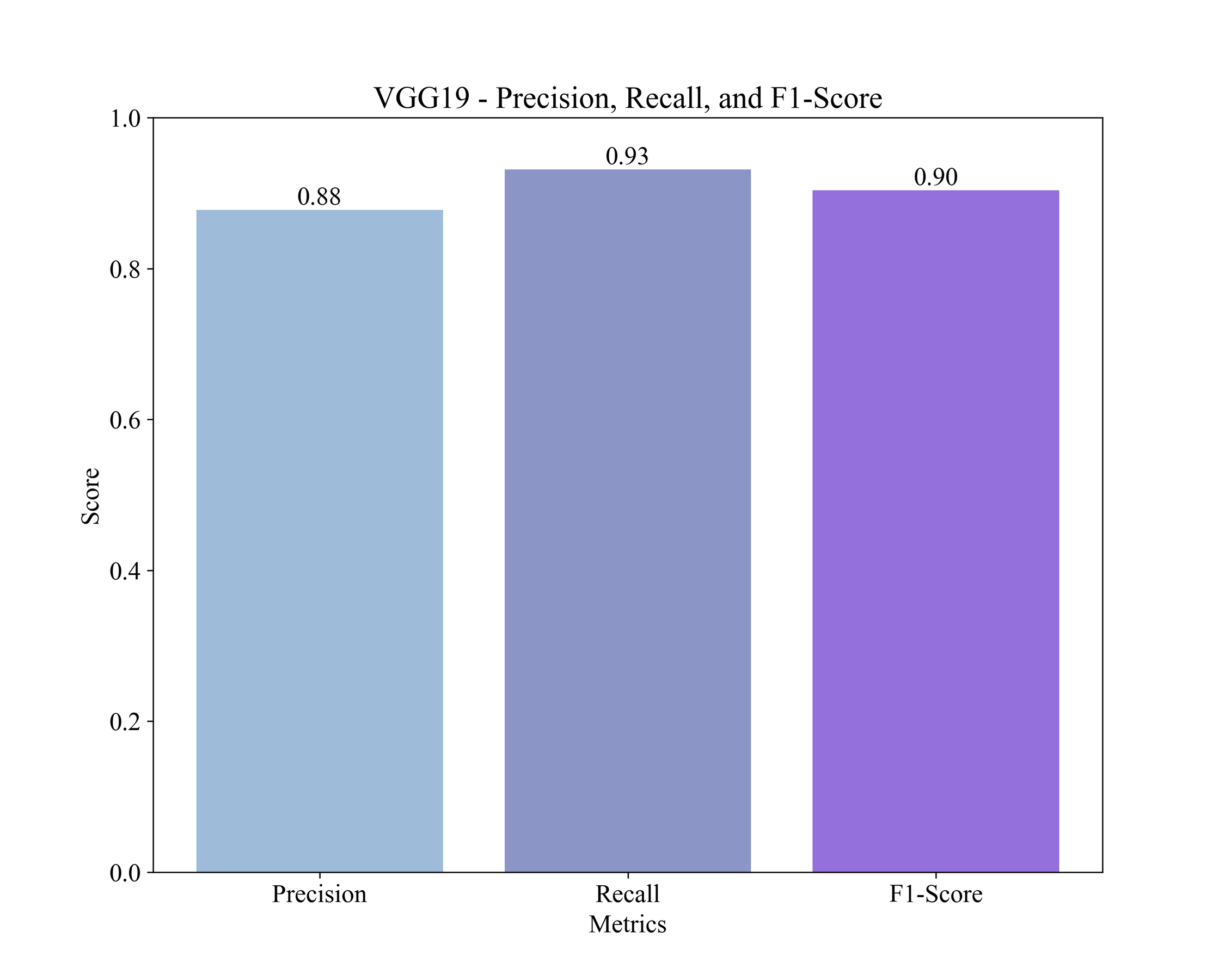


When analyzing the model’s relationship between the true positive rate and the false positive rate with the receiver operating characteristic (ROC) curve, we received an area under the curve (AUC) value of 92.0%. This finding shows that the VGG19 model can correctly distinguish between violent and nonviolent images in 92.0% of the cases (see S8 Figure).

**S8 Figure. VGG19 model receiver operating characteristic (ROC) curve.**


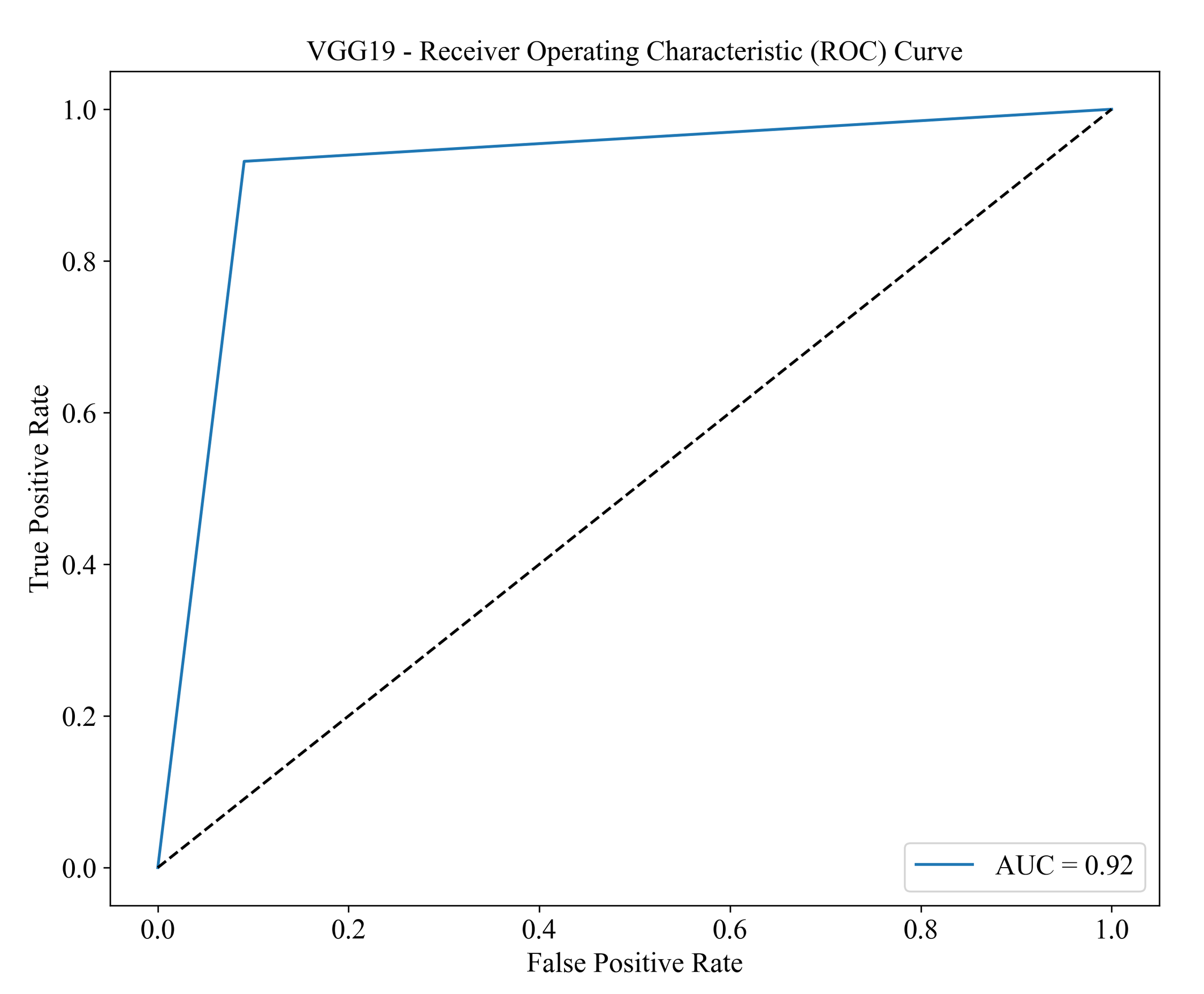


Lastly, we evaluated the VGG19 model’s performance on the test dataset in more detail by creating a confusion matrix of relative and absolute values of classified violent and nonviolent images from our test dataset (see S9 Figure and S10 Figure). The model correctly predicts nonviolent images in 91.0% of the cases (3,716 images) and violent images in 93.0% of the cases (2,657 images).

**S9 Figure. VGG19 model confusion matrix of test dataset relative values.**


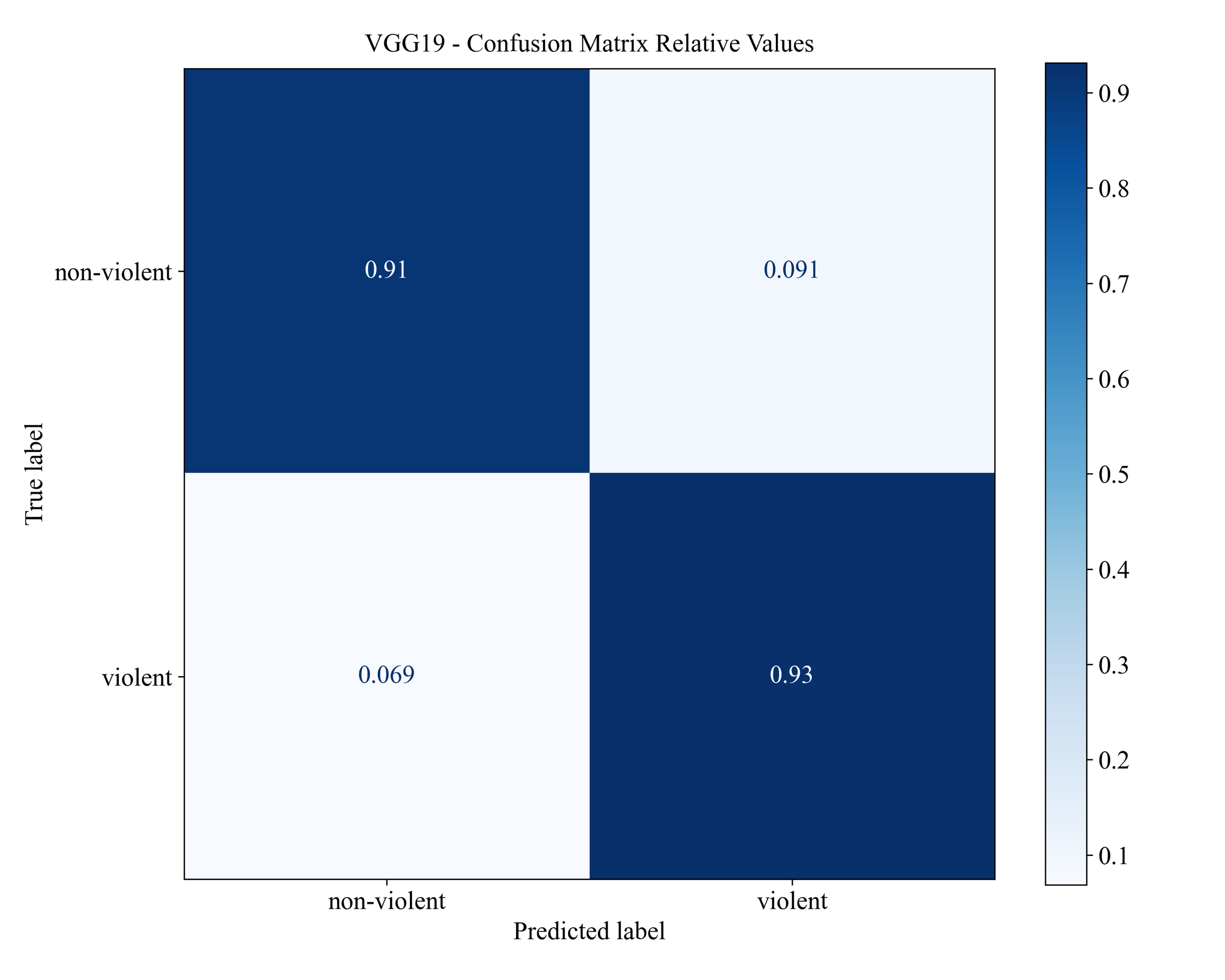


**S10 Figure. VGG19 model confusion matrix of test dataset absolute values.**


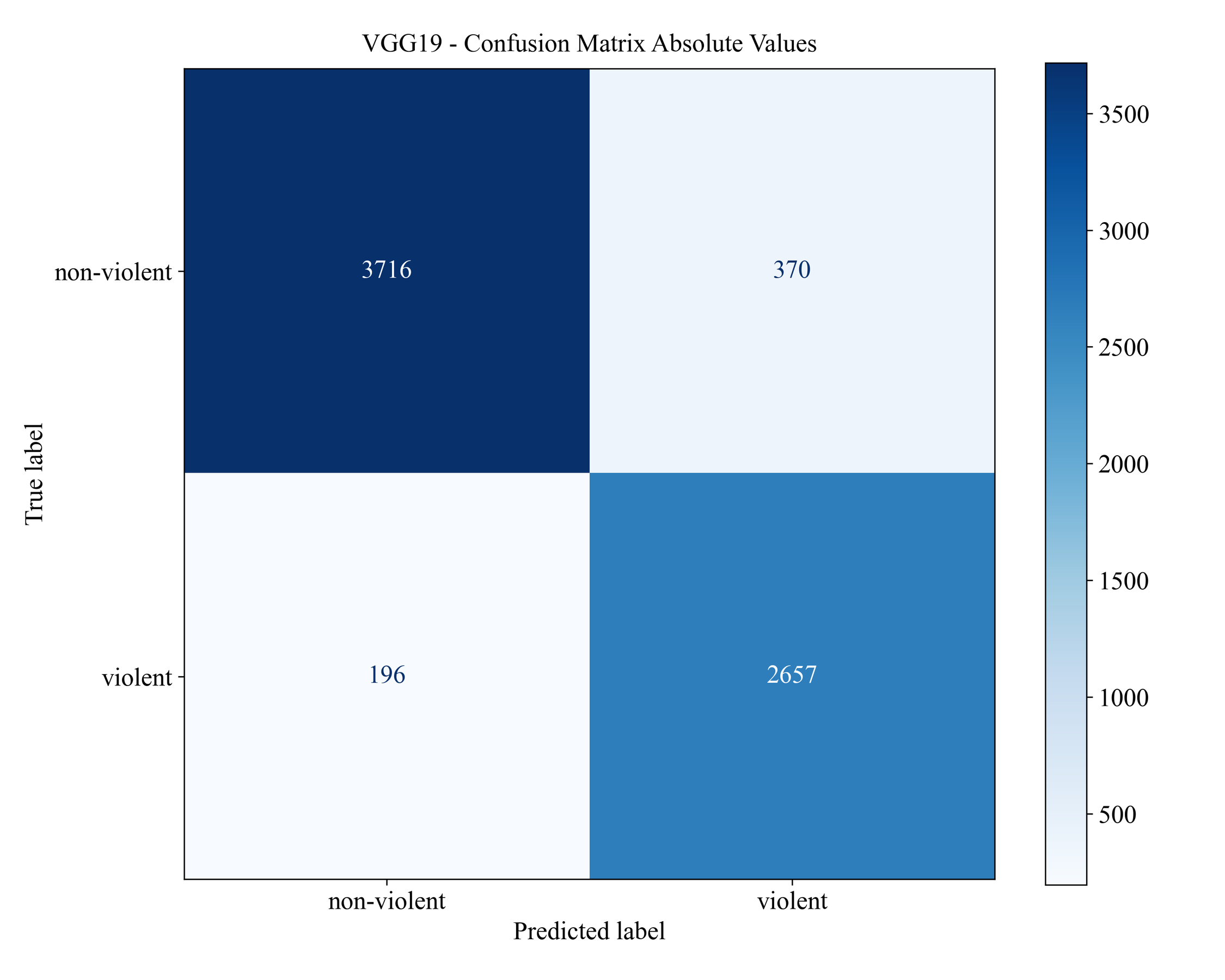


All these metrics show that the VGG19 model’s performance is sound and, thus, allows for a valid image classification within our analysis.

# **Negative binomial regression models**

To test the robustness of the results of our negative binomial regression model, we ran multiple negative binomial regression models with different datasets and variables (see S3 Table). For all models, we used the “glm.nb” function from the R package “MASS.” In the following, we will provide an overview of the different model results and explanations for diverging effects compared to the model used in the paper. Note that we did not standardize the variables before running the models, as we did not include any interaction terms and only have one numeric control variable (number of cross-posts). Likewise, standardizing the variables by subtracting the mean and dividing them by the standard deviation before running a negative binominal regression impedes the interpretability of the results. It is, therefore, often omitted when performing this type of analysis [for more information, see, e.g., 14]. Note that all other variables are held constant when interpreting the individual coefficients.

As our dependent variable is a count variable, a Poisson or negative binomial regression model is suitable for our analysis. To test whether a Poisson or negative binomial regression would be more appropriate for our data, we conducted for all models a likelihood ratio test for overdispersion in count data (“odTest” function from the R package “pscl”). The likelihood ratio test estimates whether the data’s variance equals its mean, which is the assumption of a Poisson model. The test results showed that the data’s variance was greater than the mean for all models, implying a significant data overdispersion. Thus, a negative binomial regression, which relaxes the assumption regarding variance equaling mean, was a more suitable model choice for our data [for more information, see 15]. Note that while our initial data featured a magnitude of zero comments, a zero-inflated negative binomial regression would not be a suitable model choice as this model assumes a divergent mechanism responsible for creating zero values compared to other values [for more information, see 16]. In the case of Reddit, various factors could be accountable for submissions receiving zero comments, like the novelty of the submission, its compliance with community rules, or its visibility due to cross-posts within different subreddits. Yet, as all submissions underly the same environmental conditions, no separate mechanism is responsible for creating zero comments. Also, note that the distribution of our dependent variable number of comments is very common for most social media content, as there is an abundance of content competing for user attention, reducing the likelihood for individual posts to receive much attention [see, e.g., 17].

To nonetheless test how zero comments might affect our model results, we reran the negative binomial regression with the Reddit BLM dataset, including submissions with zero comments (**model 1**). Only a few differences become visible when comparing the model 1 results with the results reported in our paper. In the following, we will mention significant coefficients that changed their effect direction or became significant/insignificant when comparing both models.

- Compared to the model reported in the paper, model 1’s “minimal traffic” coefficient is no longer significant. This change in significance can likely be attributed to the additional data added to model 1, which mainly included submissions featuring news websites with high traffic, thus overshadowing the effect of news websites with minimal traffic.
- The coefficients of r/news and r/worldnews remain significant but negatively correlated with the number of comments in model 1. This direction shift can be explained when looking into the distributions of submissions between the three subreddits and the two datasets. When excluding submissions with zero comments from the data, r/politics comprises 4,324 submissions, r/news 1,114 submissions, and r/worldnews 435 submissions. If we add submissions with zero comments to the dataset, r/politics comprises 4,593 submissions, r/news 4,052 submissions, and r/worldnews 1,084. Thus, especially r/news and r/worldnews feature submissions with zero comments, which then affect the number of comments negatively when added to the model.
- The coefficient of “NSFW” turned out to be significant. Yet, as only 15 submissions are labeled as “NSFW,” we refrain from overstating the significance of this finding due to the variable’s highly skewed distribution.
- The variable “US pacific night” is no longer significant when rerunning the model with submissions featuring zero comments. This change in significance can be explained by the fact that especially submissions posted during the morning or afternoon/evening receive zero comments. Thus, when including these submissions and rerunning the model, more noise is added that marginalizes the negative effect of submissions posted during the night.
- When comparing the coefficients of different types of news outlets between the models, the following becomes apparent: Model 1’s submissions featuring links to magazines no longer significantly correlate with the number of comments compared to submissions featuring links to newspapers, whereas submissions featuring URLs to organizations/foundations and websites have a significant negative association. Submissions featuring links to TV stations significantly positively affect the number of comments compared to submissions featuring links to newspapers. Except for websites, the coefficients of the mentioned variables only change the significance level but not the effect direction, showing that the association between the variables remains the same, yet due to different data distributions, not in a significant way. The reversed effect of submissions featuring websites on the number of comments compared to submissions featuring a link to newspapers can be explained by the added submissions with zero comments, which nearly doubled the number of submissions featuring a link to websites. Thus, in model 1, these submissions have a negative impact on the number of comments compared to submissions featuring links to newspapers.
- When comparing the model fit measure AIC between both models, model 1 performs worse than the original model included in the paper (AIC value of 60,451 compared to 50,416).

We wanted to dive deeper into the effects of a submission’s title’s sentiment by not only evaluating how positive or negative sentiment affects the number of comments but also if it matters whether Black Lives Matter (BLM) was directly addressed within the title. In line with the negativity bias [18,19], we assumed that combining a negative sentiment with mentioning BLM in a submission’s title should positively affect the number of comments. Note that we did not differentiate whether the negative sentiment directly targeted BLM, as based on the negativity bias, we assumed that negative titles generally receive more attention than positive connotated titles. We, therefore, coded the binary variable “BLM in title negative,” which is “1” if BLM appears in the submission’s title and if the BERT sentiment model detected a negative sentiment within the title. Otherwise, it was coded “0.” In 3,848 cases, the variable was coded as “0,” and in 2,025 cases, it was coded as “1.” To also test whether a positive sentiment combined with the mentioning of BLM in a submission’s title affected the number of comments, we coded another binary variable called “BLM in title positive,” which was “1” if BLM appeared in the submission’s title and the BERT sentiment model detected a positive sentiment for the tile. Otherwise, it was coded as “0.” In 5,192 cases, the variable was coded as “0,” and in 681 cases, as “1.” We reran the model with the same dataset used within the paper while dropping the BERT sentiment variable as it highly correlated with the newly added variables “BLM in title negative” and “BLM in title positive” (**model 2**). When comparing the results between model 2 and the model reported in the paper, we find the following:

- Both variables, “BLM in title negative” and “BLM in title positive,” have a significant positive effect on the number of comments. Thus, we find evidence that it does not matter whether BLM is associated with a positive or negative sentiment within a title. Instead, referencing BLM in the title positively correlates with the users’ attention. This finding shows that it is not only the sentiment of a title but also naming specific keywords like BLM that attracts the attention of users interested in news targeting BLM.
- Only marginal differences can be detected when comparing the remaining results between the two models. Compared to the model in the paper, model 2’s “political leaning liberal” coefficient and type of news outlet “website” are no longer significant. However, the direction of the coefficients remains the same, showing a similar association.
- The model fit of both models only marginally differs. Model 2 receives an AIC score of 50,386 compared to a score of 50,416 from the model used in the paper.

Lastly, we wanted to sustain the findings of our model by using a different variable to capture user attention. The only other variables available through Reddit that provide insights into how often users engaged with a submission are “score” and “upvote ratio.” The score of a submission is determined by the difference between up- and downvotes for each submission. The upvote ratio is the ratio by which submissions have been upvoted. As mentioned in our paper, up- or downvoting a submission depicts rather an indicator of whether a submission’s content aligns with the community rules of a subreddit than being an expression of attention [20]. Yet, as this information is the only available measure besides the number of comments that provides insights into user engagement, we continued with this variable as an alternative operationalization for user attention. As both upvoting and downvoting are expressions of engaging with a submission, we needed to calculate the sum of upvotes and downvotes for all submissions. Note that it would therefore be inappropriate to use the score variable as it blends both variables by subtracting them from each other. We used the “score” and “upvote ratio” variables to calculate the number of upvotes and downvotes per submission. Inspired by [21], we utilized the following formulas for receiving the number of upvotes and downvotes where $u=upvotes$, $d=downvotes$, $s=score$, $r=upvote ratio$, and $a=downvote ratio$ with the following conditions $s=u-d,$ $d=u-s$, $u=s+d$, and $a=1-r$:

Formulas for calculating the upvotes:

$$u=r\times(u+d)$$

$u=r \times(u+(u-s)$)

$$u=r \times(2u-s)$$

$$u=2ur-sr$$

$$sr=2ur-u$$

$$sr=u \times(2r-1)$$

$$u= \frac{sr}{(2r-1)}$$

Formulas for calculating the downvotes:

$$d=a \times(u+d)$$

$$d=a \times\left( (s+d \right)+d)$$

$$d= a \times(2d+s)$$

$$d=2da+sa$$

$$-sa=2da-d$$

$$-sa=d \times\left( 2a-1 \right)$$

$$d= \frac{\left( -sa \right)}{\left( 2a-1 \right)}$$

$$d= \frac{-s \times(1-r)}{(2\times\left( 1-r \right)-1}$$

After calculating upvotes and downvotes for all submissions, we added them to receive the new dependent variable “total votes.” We used this variable as an alternative operationalization of user attention. Yet, the number of comments still depicts a more substantial and reliable measure of attention (see arguments mentioned within the paper). Similar to our data reduction before, we dropped all submissions with a score of 0 and performed a negative binomial regression with “total votes” as the dependent variable in **model 3**. Most of the results of the model used in the paper could be replicated with only some effect changes, which we will mention in the following.

- In model 3, the negative sentiment of a news article’s title has a significant but negative association with the dependent variable. One possible explanation for this reversed effect could be that up- or downvoting a submission indicates whether a submission’s content aligns with the community rules. Since news titles that were classified as “negative” often contain derogatory content, they sometimes disregard the strict community rules of the subreddits. Thus, by downvoting them compared to rather positive connotated titles, users are able to sanction the submission if it does not follow the community rules.
- In model 3, the variable “political leaning conspiracy” is significantly negatively correlated with the dependent variable “total votes.” This negative relationship indicated that users did not like to up- or downvote a submission that featured a link to a news outlet with conspiratorial content compared to a news outlet with a neutral political leaning. Yet, we treated this result cautiously, as this category only comprises eight observations.
- In model 3, r/news and r/worldnews have a significant but negative effect on the dependent variable “total votes” compared to r/politics. Similarly to model 1, we detect a distributional shift in the number of submissions per subreddit. When dropping all submissions with a score of 0, r/politics comprised 4,006 submissions, r/news 3,890 submissions, and r/worldnews 1,021 submissions compared to 4,324 submissions in r/politics, 1,114 submissions in r/news, and 435 submissions in r/worldnews used within our model reported in the paper. Thus, the reduction of submissions with a score of zero does not remove as much noise (submissions with little engagement) as the reduction of submissions with zero comments. As the number of submissions in r/news and r/worldnews of model 3 is nearly three times higher compared to the data distribution within the model of the paper, the reversed effect can be attributed to a distributional shift within the data.
- In model 3, the variable “night US pacific” no longer has a significant neagtive effect compared to submissions posted during the afternoon/evening. This finding can be explained through shifts in the data distribution.
- In model 3, both types of news outlets, “organization/foundation” and “website,” have a significant negative effect compared to the reference category “newspaper.” The category “TV station,” on the other side, has a positive correlation. Compared to the original model, one possible explanation for these divergent findings is that up- or downvoting a submission indicates whether a submission’s content aligns with the community rules. As the newspapers included in our dataset are most often very prominent news outlets compared to less prominent organizations/foundations and websites, it seems plausible to assume that users rather know whether a linked newspaper aligns with the community rules of a respective subreddit compared to more unfamiliar linked organizations/foundations or websites. Therefore, we assume that users up- or downvote submissions featuring links to organizations/foundations or websites less often than submissions featuring links to newspapers. TV stations, in contrast, seem to align more with the community rules compared to newspapers (positive correlation).
- When comparing the model fit of both models, the original model outperforms model 3 by far (model 3’s AIC is 86,704, compared to 50,416).

**S3 Table. Negative binomial regression results of models with different datasets and variables (standard error in parentheses, p values in square brackets).**

|  | **model 1: complete data including zero comments** | **model 2: data without zero comments & with BLM in title variable** | **model 3: data without zero scores & total votes as dependent variable** |
| --- | --- | --- | --- |
|  | dependent count variable: number of comments | dependent count variable: number of comments | dependent count variable: total votes |
| VGG19 violent image | -0.096 (0.086) [0.264] | 0.014 (0.072) [0.845] | -0.006 (0.079) [0.936] |
| BERT sentiment negative | 0.104• (0.065) [0.035] |  | -0.283*** (0.052) [0.000] |
| BLM in title negative |  | 0.302*** (0.040) [0.000] |  |
| BLM in title positive |  | 0.212*** (0.059) [0.000] |  |
| political leaning conservative | -0.628*** (0.091) [0.000] | -0.203** (0.073) [0.006] | -0.911*** (0.089) [0.000] |
| political leaning conspiracy | -1.213 (0.776) [0.118] | -0.080 (0.990) [0.935] | -1.509* (0.724) [0.037] |
| political leaning liberal | -0.254*** (0.072) [0.001] | -0.048 (0.058) [0.406] | -0.130• (0.067) [0.051] |
| factual reporting high | 0.233*** (0.052) [0.000] | 0.091* (0.041) [0.029] | 0.565*** (0.048) [0.000] |
| factual reporting low | -0.709*** (0.136) [0.000] | -1.009*** (0.124) [0.000] | -1.246*** (0.131) [0.000] |
| traffic high | -0.541*** (0.083) [0.000] | -0.242*** (0.066) [0.000] | -0.639*** (0.079) [0.000] |
| traffic minimal | -0.200 (0.269) [0.459] | -1.221*** (0.217) [0.000] | -0.665** (0.257) [0.010] |
| subreddit news | -0.756*** (0.051) [0.000] | 0.996*** (0.048) [0.000] | -1.064*** (0.048) [0.000] |
| subreddit worldnews | -0.299*** (0.076) [0.000] | 0.619*** (0.071) [0.000] | -0.852*** (0.070) [0.000] |
| NSFW | -1.399* (0.586) [0.017] | -1.174 (0.798) [0.141] | -0.447 (0.525) [0.395] |
| link flair | -0.317*** (0.049) [0.000] | -0.569*** (0.037) [0.000] | -0.814*** (0.046) [0.000] |
| number of cross-posts | 1.377*** (0.022) [0.000] | 0.901*** (0.014) [0.000] | 1.732*** (0.019) [0.000] |
| weekend US pacific | 0.394*** (0.054) [0.000] | 0.237*** (0.042) [0.000] | 0.534*** (0.051) [0.000] |
| morning US pacific | -0.004 (0.049) [0.940] | -0.016 (0.039) [0.691] | -0.021 (0.046) [0.643] |
| night US pacific | 0.070 (0.063) [0.262] | -0.085• (0.050) [0.090] | 0.081 (0.058) [0.167] |
| type of news outlet magazine | 0.142 (0.115) [0.215] | 0.251** (0.086) [0.003] | 0.380*** (0.108) [0.000] |
| type of news outlet news agency | -0.252• (0.133) [0.058] | -0.741*** (0.102) [0.000] | -0.495*** (0.124) [0.000] |
| type of news outlet organization/foundation | -0.736** (0.236) [0.002] | -0.186 (0.182) [0.307] | -0.862*** (0.227) [0.000] |
| type of news outlet radio | -0.350• (0.197) [0.076] | -0.338* (0.154) [0.028] | -0.761*** (0.179) [0.000] |
| type of news outlet TV station | 0.475*** (0.057) [0.000] | 0.044 (0.047) [0.341] | 0.139* (0.054) [0.010] |
| type of news outlet website | -0.258*** (0.064) [0.000] | 0.061 (0.052) [0.236] | -0.263*** (0.060) [0.000] |
| Constant | 3.358*** (0.121) [0.000] | 3.218*** (0.089) [0.000] | 6.067*** (0.113) [0.000] |
| Observations | 9,729 | 5,873 | 8,917 |
| Null deviance | 15,007.1 on 9,728 df | 14,208.2 on 5,872 df | 21,675 on 8,916 df |
| Res. deviance | 9,859.7 on 9,705 df | 6,894.7 on 5,848 df | 11,726 on 8,893 df |
| AIC | 60,451 | 50,386 | 86,704 |
| Theta | 0.213 | 0.561 | 0.263 |
| Std. Error | 0.003 | 0.009 | 0.003 |
| Note: •p<0.1; *p<0.05; **p<0.01, ***p<0.001 | | | |

Thus, when comparing all three models with our original model reported in the paper, the main effects remain robust with minor but explainable deviations. This finding supports the robustness of the model and its results presented in our paper. Likewise, the model reported in the paper exhibits an equal or substantially better model fit than the other models, again highlighting the model’s suitability for our analysis.

# **References**

1. Won D, Steinert-Threlkeld ZC, Joo J. Protest activity detection and perceived violence estimation from social media images. MM 2017 - Proceedings of the 2017 ACM Multimedia Conference. 2017. pp. 786–794. doi:10.1145/3123266.3123282

2. Hamborg F, Donnay K. NewsMTSC: a dataset for (multi-)target-dependent sentiment classification in political news articles. Proceedings of the 16th Conference of the European Chapter of the Association for Computational Linguistics. 2021. pp. 1663–1675. doi:10.5167/uzh-207183

3. Rucht D. Violence and New Social Movements. International Handbook of Violence Research. Dordrecht: Springer Netherlands; 2003. pp. 369–382. doi:10.1007/978-0-306-48039-3_20

4. wondonghyeon. Protest Activity Detection and Perceived Violence Estimation from Social Media Images. In: github [Internet]. 2018 [cited 5 Jun 2023]. Available from: https://github.com/wondonghyeon/protest-detection-violence-estimation

5. fhamborg. NewsMTSC dataset. In: Hugging Face [Internet]. 2022 [cited 6 Jun 2023]. Available from: https://huggingface.co/datasets/fhamborg/news_sentiment_newsmtsc

6. Hugging Face. bert-base-uncased. In: Hugging Face [Internet]. 2023 [cited 2 Jun 2023]. Available from: https://huggingface.co/bert-base-uncased

7. Devlin J, Chang M-W, Lee K, Toutanova K. BERT: Pre-training of Deep Bidirectional Transformers for Language Understanding. arXiv; 2019. doi:10.48550/arXiv.1810.04805

8. Horne BD, Adalis S. The Impact of Crowds on News Engagement: A Reddit Case Study. The Workshops of the Eleventh International AAAI Conference on Web and Social Media. AAAI Technical Report; 2017. pp. 751–758. doi:10.1609/icwsm.v11i1.14977

9. Ridnik T, Ben-Baruch E, Noy A, Zelnik-Manor L. ImageNet-21K Pretraining for the Masses. arXiv; 2021. doi:10.48550/arXiv.2104.10972

10. Tan M, Le Q. Efficientnet: Rethinking model scaling for convolutional neural networks. International conference on machine learning. PMLR; 2019. pp. 6105–6114.

11. Li Z, Liu F, Yang W, Peng S, Zhou J. A survey of convolutional neural networks: analysis, applications, and prospects. IEEE transactions on neural networks and learning systems. 2022;33: 6999–7019. doi:10.1109/TNNLS.2021.3084827

12. Dosovitskiy A, Beyer L, Kolesnikov A, Weissenborn D, Zhai X, Unterthiner T, et al. An image is worth 16x16 words: Transformers for image recognition at scale. arXiv; 2020. doi:10.48550/arXiv.2010.11929

13. Han K, Wang Y, Chen H, Chen X, Guo J, Liu Z, et al. A survey on vision transformer. IEEE transactions on pattern analysis and machine intelligence. 2022;45: 87–110. doi:10.1109/TPAMI.2022.3152247

14. Cameron AC, Trivedi PK. Regression analysis of count data. Cambridge university press; 2013.

15. Hilbe JM. Negative binomial regression. Cambridge University Press; 2011.

16. Yau KK, Wang K, Lee AH. Zero-inflated negative binomial mixed regression modeling of over-dispersed count data with extra zeros. Biometrical Journal: journal of mathematical methods in biosciences. 2003;45: 437–452. doi:10.1002/bimj.200390024

17. Feng L, Hu Y, Li B, Stanley HE, Havlin S, Braunstein LA. Competing for attention in social media under information overload conditions. PloS one. 2015;10: e0126090. doi:10.1371/journal.pone.0126090

18. Shoemaker PJ. Hardwired for news: Using biological and cultural evolution to explain the surveillance function. Journal of Communication. 1996;46: 32–47. doi:10.1111/j.1460-2466.1996.tb01487.x

19. Rozin P, Royzman EB. Negativity bias, negativity dominance, and contagion. Personality and Social Psychology Review. 2001;5: 296–320. doi:10.1207/S15327957PSPR0504_2

20. Reddit. Reddiquette [Internet]. 2021 [cited 30 Apr 2022]. Available from: https://reddit.zendesk.com/hc/en-us/articles/205926439-Reddiquette

21. How to calculate individual upvotes and downvotes of a post (Approximate). In: r/TheoryOfReddit [Internet]. 2018. Available from: www.reddit.com/r/TheoryOfReddit/comments/a0yt70/how_to_calculate_individual_upvotes_and_downvotes/
